# RIBEX: predicting and explaining RNA binding across structured and intrinsically disordered regions (IDR)-rich proteins

**DOI:** 10.1093/bioinformatics/btag471

**Published:** 2026-08-21

**Authors:** Samuele Firmani, Felix Steinbauer, Gjergji Kasneci, Annalisa Marsico, Marc Horlacher

**Affiliations:** Computational Health Center, Helmholtz Center Munich, Neuherberg 85764, Germany; School of Computation, Information and Technology, Technical University of Munich, Munich 80333, Germany; School of Computation, Information and Technology, Technical University of Munich, Munich 80333, Germany; Munich Center for Machine Learning (MCML), Munich, Germany; School of Computation, Information and Technology, Technical University of Munich, Munich 80333, Germany; Munich Center for Machine Learning (MCML), Munich, Germany; School of Social Sciences and Technology, Technical University of Munich, Munich 80333, Germany; Computational Health Center, Helmholtz Center Munich, Neuherberg 85764, Germany; Cluster for Nucleic Acid Sciences and Technologies—NUCLEATE, Munich, Germany; Computational Health Center, Helmholtz Center Munich, Neuherberg 85764, Germany; School of Computation, Information and Technology, Technical University of Munich, Munich 80333, Germany

## Abstract

**Motivation:**

RNA-binding proteins (RBPs) regulate post-transcriptional processes, yet many remain undiscovered because RNA-binding activity often occurs outside canonical RNA-binding domains (RBDs), including within intrinsically disordered regions (IDRs) or through protein complexes. Computational methods can help identify novel RBPs, but approaches relying solely on sequence-derived features or ignoring the cellular interaction context are limited in capturing the complexity of RNA-binding behavior. To date, no framework rigorously integrates both sequence information and protein interaction context for RBP prediction.

**Results:**

We introduce RIBEX, a multimodal framework that combines protein language model (pLM) embeddings with protein interactome topology to improve RBP prediction and interpretation. Specifically, we integrate sequence representations with graph-derived positional encodings (PE) from the human STRING protein–protein interaction (PPI) network. PE are computed using Personalized PageRank, reduced with principal component analysis, and fused with pooled sequence embeddings through FiLM conditioning, while Low-Rank Adaptation (LoRA) enables parameter-efficient task adaptation. Across both an annotation-based benchmark and experimental RNA Interactome Capture (RIC) dataset, PE consistently improves predictive performance, indicating that interactome topology provides complementary information beyond sequence features. LoRA adaptation of ESM2-650M further yields larger gains than simply scaling frozen backbone size. RIBEX outperforms state-of-the-art methods such as RBP-TSTL and HydRA, particularly on challenging subsets including proteins lacking canonical RBDs and those enriched in IDRs. For interpretability, we combine sequence-level computational alanine scanning with network-level positional-encoding ablation and inverse-PCA mapping, recovering known RNA-binding domains, IDR-associated contributions, and functional interactome communities linked to RBP predictions.

## 1 Introduction

The regulation of RNA transcripts is orchestrated by a diverse class of molecules known as RNA-Binding Protein (RBPs). Traditionally, RBPs were defined by canonical RNA-Binding Domain (RBDs), however, high-throughput methods like RNA Interactome Capture (RIC) uncovered a large “hidden” layer of the interactome (Castello *et al*. 2013), revealing hundreds of previously unrecognized and non-canonical RBPs. Many of these lack classical RBDs and instead rely on low-complexity, Intrinsically Disordered Region (IDRs), now recognized as key mediators of RBPs–RNA interactions (Hentze *et al.* 2018).

Although RBPs have been systematically annotated across multiple species in diverse databases their repertoire remains far from complete. RIC enables RBP identification on a large scale, but it provides only a condition-specific snapshot of RNA–protein interactions, often missing low-abundance RBPs, proteins that bind non-polyadenylated RNAs, and RBPs expressed in other cell types or experimental contexts (e.g. a RIC screen in HEK293 cells will not detect RBPs restricted to neurons or liver). Computational methods that learn the sequence and structural “grammar” of binding can help bridge these gaps by enabling condition-independent, proteome-wide RBP prediction where experimental data are unavailable. However, novel RBP prediction remains challenging because many RBPs lack the evolutionary conservation or structural signatures used by classical methods.

Early computational RBP predictors relied mainly on known RBDs, sequence motifs, evolutionary profiles, or hand-crafted sequence and structural features. However, high-throughput RBP discovery showed that many experimentally detected RBPs lack recognizable canonical RNA-binding homology, motivating methods that capture weaker or non-canonical sequence signals (Castello *et al*. 2012). TriPepSVM addressed this by modeling short amino-acid motifs, especially tri-peptides, and showed strong performance for RBPs with disordered regions, where basic- and aromatic-rich motifs are enriched among known binders (Bressin *et al*. 2019). However, purely sequence-based approaches remain limited when long-range interactions are essential for protein function.

A large class of pretrained Protein Language Model (pLMs), such as ESM-2/3 (Lin *et al*. 2022), treat amino acid sequences like natural language and are trained self-supervised on large protein sequence datasets. They capture contextual, local, and global sequence features, as well as latent structural and functional information, making them the leading framework for transfer learning in protein property and structure prediction (comprehensive overview in (Xiao *et al*. 2025)). A landmark study, Support Vector Machine obtained from neighborhood associated RBPs (RBPTSTL) (Peng *et al*. 2022), introduced a two-stage transfer learning approach to improve genome-scale RBP prediction. It leverages high-dimensional embeddings from pLMs with limited task-specific data: a pre-trained ESM model first extracts generic sequence features, which are then fine-tuned on annotated RBPs to capture RNA-binding-specific signatures.

However, even pLM-based models are limited because they mainly learn the internal context of individual sequences and often overlook extrinsic cellular context. RBPs cluster in functional interactome neighborhoods, such as the spliceosome or ribosome, and a protein’s RNA-binding propensity is often reflected in its PPI network. Tools such as Support Vector Machine obtained from neighborhood associated RBPs (SONAR) (Brannan *et al*. 2016) were among the first to demonstrate that analyzing PPI association patterns could discover novel RBPs that sequence-only methods missed. More recently, Hybrid Ensemble Classifier for RNA-Binding Proteins (HydRA) (Jin *et al*. 2023) integrated this interaction context with deep learning, proving that the “neighborhood” of a protein provides a powerful signal for its RNA-binding capacity. Despite these advancements, a gap remains for non-canonical RBPs, and current models often fail to capture the synergy between a protein’s global network position and its local, disordered binding motifs.

RIBEX therefore uses graph-derived positional encodings (Positional Encoding (PEs)) to summarize each protein’s topological role in the PPI network, aiming to distinguish intrinsically disordered proteins near known RBPs—which are more likely functional binders—from proteins with similar sequences but different interactome roles.

In this work, we present RIBEX, a simple yet powerful multimodal framework integrating protein language modeling with network context for improved RNA-binding prediction. By combining parameter-efficient Low-Rank Adaptation (LoRA) fine-tuning of ESM-2 with PPI-derived positional encodings, RIBEX merges intrinsic sequence features with extrinsic interactome context. This preserves the biological knowledge encoded in foundation models while improving detection of non-canonical and IDR-mediated RBPs, outperforming state-of-the-art methods such as RBP-TSTL and HydRA.

We further interpret predictions using *in silico* alanine scanning (Kortemme *et al*. 2004) to identify key sequence regions—such as IDRs or specific domains—driving RNA-binding predictions. These signals align with structural confidence patterns from AlphaFold, supporting the biological plausibility of our approach. Beyond predictive accuracy and interpretability, our work clarifies when task-specific adaptation is beneficial and highlights the complementary value of network information in uncovering functionally relevant RBPs.

## 2 Materials and methods

We formulate RBP identification as a binary classification task over human proteins, using (i) the amino-acid sequence of each protein and (ii) its topological context in a human PPI graph derived from STRING (Szklarczyk *et al*. 2023). In brief, RIBEX first encodes the protein sequence with a pLM, separately summarizes each protein’s STRING neighbourhood with PPR-based positional encodings, and then combines both streams with a FiLM layer before RNA-binding classification.

### 2.1 Data collection and pre-processing

We use three datasets as starting points: (a) The Bressin dataset (used in (Bressin *et al*. 2019) for TriPepSVM) is based on the UniprotKB database (Uniprot Consortium 2025) and uses QuickGO (Binns *et al*. 2009) to annotate RNA-binding behavior. (b) The RIC (Hentze *et al.* 2018) contains experimentally determined *in vivo* RNA–protein interactions. (c) The HydRA dataset (Jin *et al*. 2023) combines protein sequence information and protein interaction context features. For more details on the original datasets, see Supplemental Section A.1.1, available as supplementary data at *Bioinformatics* online.

After collecting the source datasets, we apply several *pre-processing* steps. We restrict the analysis to the human proteome (taxon ID 9606) and filter accordingly. All protein sequences are truncated at 1024 residues (as described in (Peng *et al*. 2022)) and transformed into dense embeddings using ESM-2 (650M and 3B variants) (Lin *et al*. 2022) as well as ProtT5-XL (3B) (Elnaggar *et al*. 2022). Only proteins for which embeddings are available across all compared pLMs are retained, forming a shared gene pool that ensures model-agnostic and directly comparable evaluation. For the HydRA dataset specifically, we additionally evaluate (see Fig. 2) on a variant of the full dataset restricted to proteins containing no known binding domains. This non-RBD subset allows us to assess model performance on harder-to-classify sequences that lack known binding domains. For more details on this dataset variant, see Supplemental Section A.1.1, available as supplementary data at *Bioinformatics* online. To enable our interpretability analysis (see Section 2.5 and Supplemental Section C, available as supplementary data at *Bioinformatics* online) and to verify the RNA-binding class labels, we collect additional metadata in the form of the RNA-binding flag (QuickGO: 0003723) from QuickGO and domain annotations for IDRs and other functional regions from InterPro (Blum *et al*. 2025) and MobiDB (Piovesan *et al*. 2025).

To avoid inflated performance caused by evaluating on proteins similar to those in the training set—*homology leakage*—we partition the dataset into training, validation, and test sets at the level of *homology clusters* rather than individual sequences. These clusters are obtained with MMseqs2 (Steinegger and Söding 2017) applied to full sequences using a minimum sequence identity of 20% and bidirectional coverage ≥50%. Singleton proteins were retained as one-member clusters. Each cluster was then assigned as an indivisible unit to exactly one partition, preventing close homologs from being shared between training, validation, and test sets. This identity cutoff is stricter than those used in previous RBP benchmarks (Supplemental Section A.1.2, available as supplementary data at *Bioinformatics* online). For the RIC and Bressin benchmarks, the cluster-aware split allocates 90% of proteins to training and reserves 10% as hold-out data; the hold-out is divided into validation (one third, early stopping/epoch selection) and test (two thirds, final evaluation); partition sizes and class compositions are listed in Supplemental Table 1, available as supplementary data at *Bioinformatics* online. HydRA comparisons use the published HydRA test pool with a separate validation/test subdivision as described in Supplemental Section A.1.3 and A.2, available as supplementary data at *Bioinformatics* online; see Supplemental Table 2, available as supplementary data at *Bioinformatics* online for partition sizes.

### 2.2 Model architecture

**Figure 1:**
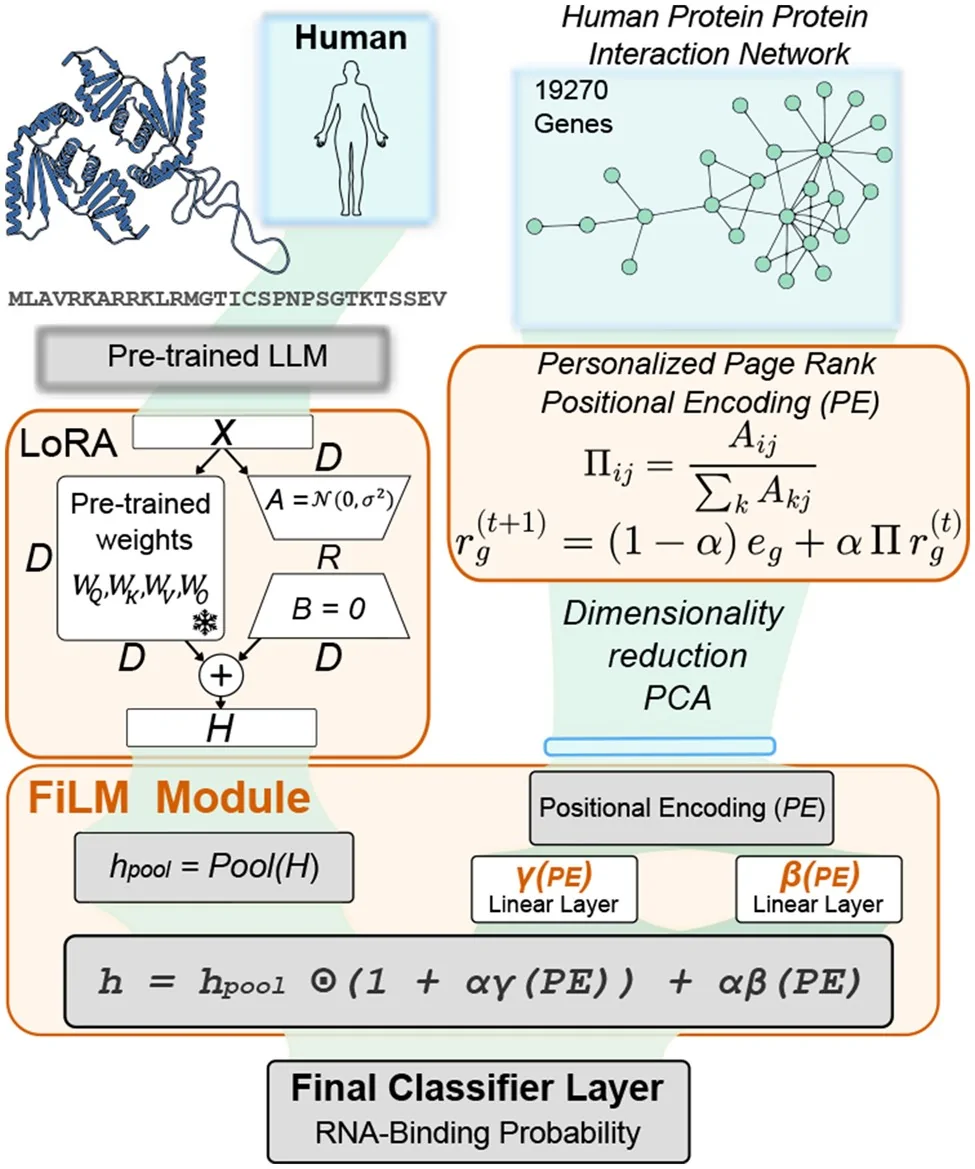
Overview of RIBEX. A protein language model encodes the amino-acid sequence, while graph-derived positional encodings from the human STRING PPI network (Personalized PageRank (PPR) followed by Principal Component Analysis (PCA)) capture interactome context. A FiLM layer conditions the pooled sequence representation on the positional encoding, and a classifier predicts RNA-binding probability.

RIBEX integrates sequence-based and network-derived node positions to predict human RNA-binding proteins. A pre-trained pLM first encodes the primary sequence into high-dimensional, contextualized residue embeddings. These features are subsequently condensed via masked mean pooling to generate a fixed-length protein representation. To ensure computational efficiency, we employ LoRA (Hu *et al*. 2021) for model fine-tuning. While the core pLM weights remain frozen, trainable low-rank matrices are integrated into the attention layers and optimized for the classification task (Fig. 1). Concurrently, task-specific layers for feature fusion (Section 2.3) and final prediction are trained in an end-to-end fashion.

### 2.3 Positional encoding and feature integration

Beyond sequence data, we incorporate the broader network context of each protein through its position in the human PPI graph. We characterize the local topology of each node using PPR vectors (Page *et al*. 1999). Starting from a weighted adjacency matrix *A* derived from STRING (Szklarczyk *et al*. 2023), we define the column-stochastic transition matrix $\Pi_{ij}=A_{ij}/\sum_{k}A_{kj}$. For a protein index *g*, we define a restart vector $e_{g}\in R^{N}$, where ${\left(e_{g}\right)}_{i}=1$ if i=g and zero otherwise. The corresponding PPR vector is estimated iteratively as $r_{g}^{\left(t+1\right)}=(1-\alpha )e_{g}+\alpha \Pi r_{g}^{\left(t\right)}$ until convergence. The resulting vector $r_{g}$ captures the steady-state visitation probabilities of random walks with restart from node *g*. Stacking these vectors across the interactome yields a high-dimensional positional representation. Because the raw PPR dimensionality is too large for direct model input, we reduce it with PCA before training, retaining $d_{\text{PE}}$ principal components; the chosen $d_{\text{PE}}$ for each model and benchmark is reported in Supplementary Tables 3 and 4, available as supplementary data at *Bioinformatics* online. To fuse sequence and graph-derived features, we use a Feature-wise Linear Modulation (FiLM) layer (Perez *et al*. 2018). Two learned projections of the reduced positional encoding (PE) generate a feature-wise scaling term γ(PE) and shift term β(PE). Given the pooled sequence feature vector *x*, we apply a residual FiLM transformation h=x⊙(1+αγ(PE))+αβ(PE), where ⊙ denotes element-wise multiplication and α is a learned scalar. The modulated representation is then passed to a classifier head to estimate the RNA-binding probability. This head is identical in the LoRA and FiLM settings: LayerNorm(H)→Dropout(0.2)→Linear(H,2), where *H* is the pooled sequence-feature dimension. The linear layer outputs two logits (RBP vs non-RBP), converted to class probabilities with a softmax.

### 2.4 Training procedure

**Figure 2:**
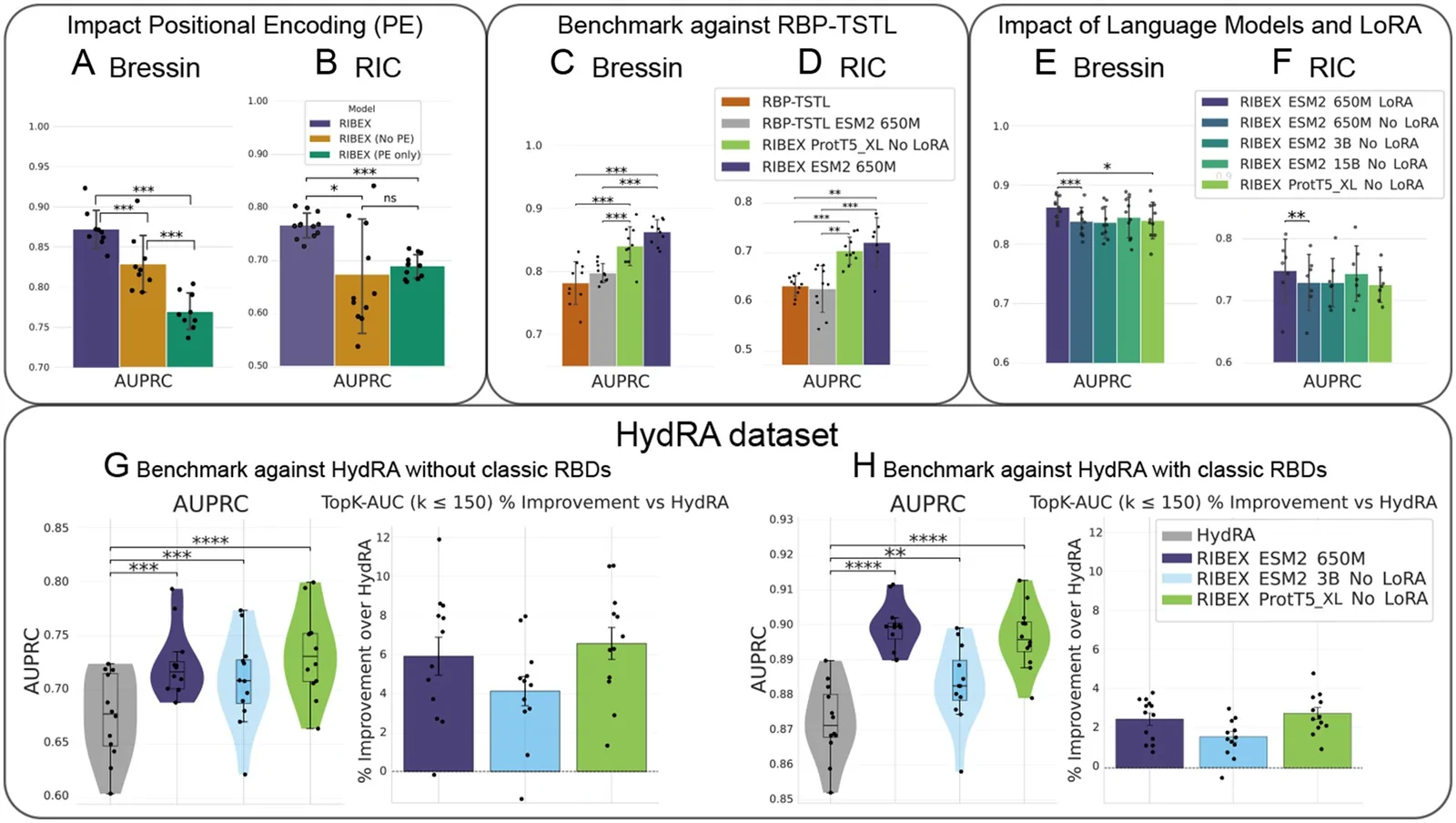
Benchmark performance of RIBEX across datasets and settings. (A–B) Effect of PPI-derived positional encodings (PEs) on the Bressin dataset (A) and the RIC dataset (B). (C–D) Comparison with RBP-TSTL on the Bressin dataset (C) and the RIC dataset (D). (E–F) Effect of pLM backbone choice and LoRA fine-tuning on the Bressin dataset (E) and the RIC dataset (F). (G–H) Comparison with HydRA on proteins without classical RNA-binding domains (G) and with classical RNA-binding domains (H); panels report AUPRC and the area under the Precision-at-top-*K* curve (Area Under the Precision-at-Top-*K* Curve (TopK-AUC), k≤150). Bars summarize runs/splits; asterisks denote paired two-sided *t*-test significance on matched seeds/splits (*P<0.05, **P<0.01, ***P<0.001).

We train the model for binary classification using a class-weighted loss to account for label imbalance. In the LoRA setting, optimization updates the LoRA adapter parameters together with the task-specific FiLM and classification layers, while the frozen pLM backbone remains unchanged. Hyperparameter optimization was conducted via random search over the training hyperparameter space; the final settings used for each model and benchmark are summarised in Supplementary Tables 3 and 4, available as supplementary data at *Bioinformatics* online. To prevent overfitting and ensure an unbiased evaluation, we employ early stopping optimized for the Area Under the Precision–Recall Curve (AUPRC) on an internal validation set. Final performance metrics are subsequently reported exclusively on a strictly held-out test partition. To guarantee robustness across random initializations, we evaluate the model using repeated random sub-sampling validation (Monte Carlo cross-validation) over multiple independent seeds. For pairwise benchmark comparisons shown in Fig. 2, statistical significance is assessed with paired two-sided *t*-tests on matched seeds/splits (i.e. paired runs sharing the same train/test partition). Benchmark comparisons against HydRA (Jin *et al*. 2023) are additionally carried out on the curated non-RBD subset of the original HydRA test set (see Supplemental Section A.1.1, available as supplementary data at *Bioinformatics* online), together with Precision at top *K* as an additional retrieval metric; the full evaluation protocol is described in Supplemental Section A.2, available as supplementary data at *Bioinformatics* online.

### 2.5 Model interpretation

**Figure 3:**
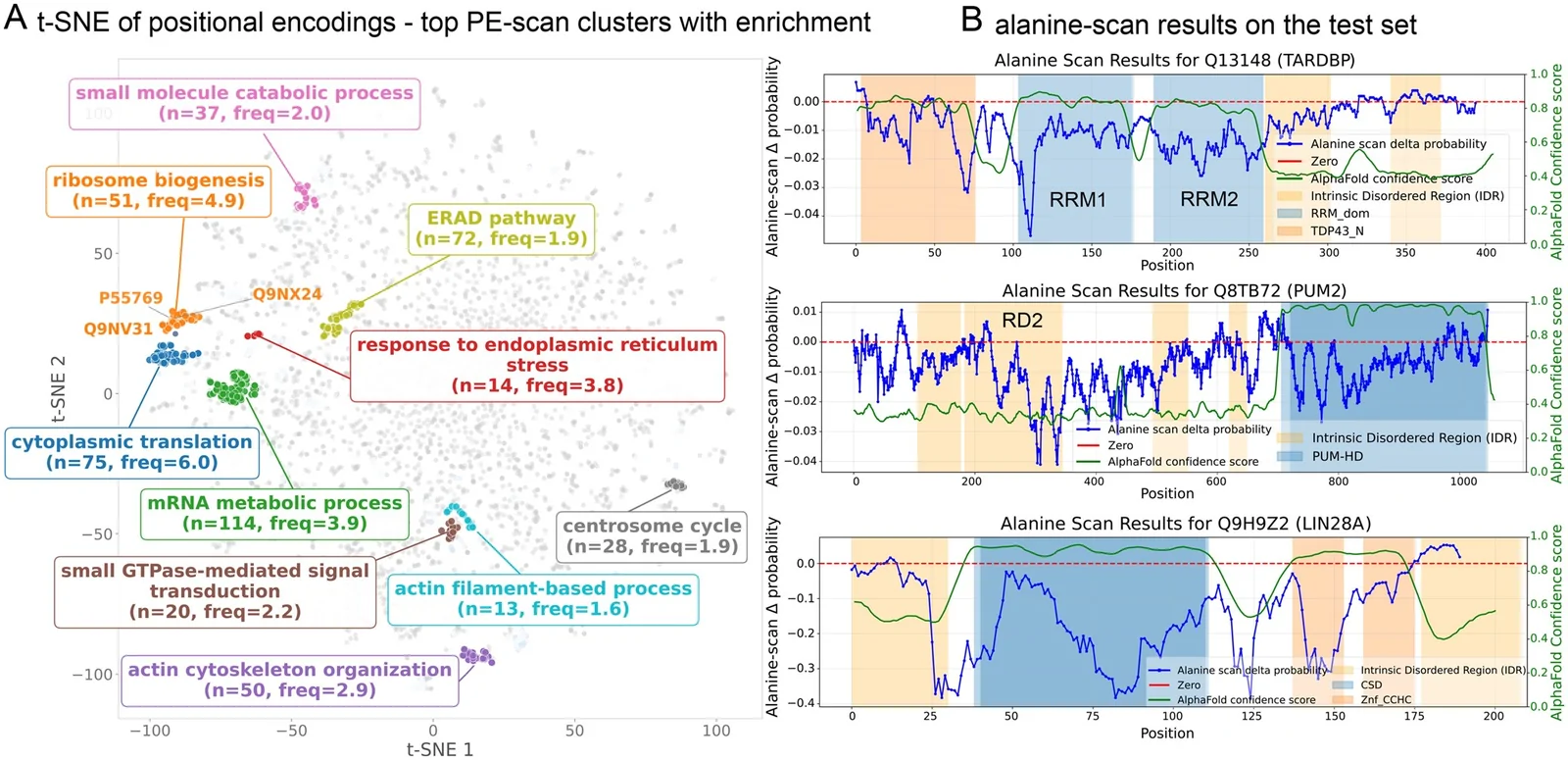
Explainability analysis of RIBEX predictions. (A) DBSCAN clustering of influential nodes in the t-SNE space. Colors indicate clusters; the legend reports cluster size (*n*), mean perturbation frequency (freq), and the top enriched Gene Ontology (GO) biological-process term. Labels highlight representative model-derived RNP-complex partner nodes, including IMP3 (Q9NV31), SNU13/NHP2L1 (P55769), and NHP2 (Q9NX24). (B) Sequence-level computational alanine scans for LIN28A, PUM2, and TARDBP/TDP-43. The blue trace shows the per-window probability change $\Delta {\hat{y}}_{\text{Ala}}$, the red line marks the zero baseline, and the green trace shows smoothed AlphaFold pLDDT. Colored tracks indicate InterPro domain annotations and MobiDB IDRs.

For the *sequence-level explanation via in silico Alanine scanning* (Kortemme *et al*. 2004) of any given protein sequence, we first estimate its baseline RNA-binding probability, denoted $\hat{y}$. Then, we systematically substitute sliding windows of ten consecutive residues with alanine, advancing one residue at a time (stride 1). The fine-tuned language model is subsequently re-evaluated on each modified sequence, giving ${\hat{y}}_{\text{Ala}}$. We measure the impact of the perturbation for each sequence window by the change in predicted probability $\Delta {\hat{y}}_{\text{Ala}}={\hat{y}}_{\text{Ala}}-\hat{y}$. A pronounced drop in the assigned probability (a highly negative $\Delta {\hat{y}}_{\text{Ala}}$) implies that the originally present residues are critical for conferring RNA-binding potential. Throughout this procedure, the positional encoding (PE) is held fixed at its original value, ensuring that any change in predicted probability stems from the sequence perturbation alone. Besides the sequence-level analysis, we investigate how the graph-derived positional encodings affect the model’s predictions (Fig. 3). Keeping the amino acid sequence fixed, we perform *network-level feature ablation* by sequentially zeroing out individual dimensions of the reduced PE vector and measuring the resulting shift in the prediction score $\Delta {\hat{y}}_{\text{PE},j}=P(y|x,p^{\left(j\right)})-P(y|x,p)$, where $p\in R^{d}$ denotes the original PE vector, $p^{\left(j\right)}$ represents the same vector with its *j*-th dimension zeroed out, *x* is the input amino acid sequence, and P(y∣·) is the probability estimate from the trained classification network. PE dimensions whose ablation substantially decreases binding probability ($\Delta {\hat{y}}_{\text{PE},j}<-0.01$) are highlighted as positive predictive evidence for RNA interaction. Because each reduced PE dimension is a PCA component, its effect is not directly interpretable at the level of individual interaction partners. For each scanned protein, we therefore collect the PE dimensions whose ablation decreases RNA-binding probability beyond this threshold, set them to zero simultaneously, and apply the inverse PCA transform. This yields two reconstructed vectors in the full PPR-derived node space, before and after ablation, corresponding to scores over all proteins in the STRING interactome. We then compute the absolute node-wise difference between the original and ablated reconstructions and rank all nodes accordingly. For each protein, we retain the top 40 nodes with the largest differences and interpret them as those most affected by the removed PE signal. Across proteins, we aggregate how often each node appears in these top-40 lists, producing a frequency-based map of interactome regions recurrently supporting positive predictions. For visualization, these aggregated node frequencies are overlaid on a t-distributed Stochastic Neighbor Embedding (t-SNE) embedding of the reduced PE matrix for all STRING proteins (Fig. 3) and Fig. S4, available as supplementary data at *Bioinformatics* online.

## 3 Results

### 3.1 Impact of design choices

To disentangle the *contribution of the pLM* from that of parameter-efficient adaptation, we compared RIBEX with LoRA on ESM2-650M against RIBEX without LoRA using ESM2-650M, ESM2-3B, ESM2-15B, and ProtT5-XL backbones (Fig. 2E for the Bressin dataset; Fig. 2F for the RIC dataset). Across both datasets, adding LoRA to ESM2-650M significantly improves performance over the untuned model, with the largest gains in Matthews Correlation Coefficient (MCC) (Supplemental Fig. 6E, F, available as supplementary data at *Bioinformatics* online) and AUPRC. In contrast, varying the pretrained backbone and scaling model size without LoRA yields only modest differences: a slight trend appears on the Bressin dataset but is weak and does not transfer clearly to the more challenging RIC benchmark. All in all, within this controlled comparison, task-specific LoRA adaptation of ESM2-650M contributes more clearly to performance than increasing the size of the frozen backbone alone; exploratory LoRA runs on larger ESM2 backbones are discussed in Supplemental Section A.3, available as supplementary data at *Bioinformatics* online. This pattern is consistent with recent analyses showing that, although pretrained protein language models outperform naive sequence representations, performance on many downstream tasks does not reliably scale with pretraining/model size and often depends on low-level features learned early in pretraining (Li *et al*. 2024).

To assess the *contribution of positional encodings (PEs)*, we compare RIBEX with and without PEs and with a PE-only variant in two benchmark settings. In panel A of Fig. 2, corresponding to the Bressin dataset, the PE-enabled model consistently outperforms the no-PE variant. The same trend is observed in panel B on the human RIC dataset, where adding PEs to the LoRA-fine-tuned human model yields higher scores across the reported evaluation metrics. The PE-only model is below sequence-only on the Bressin dataset but approaches it on RIC, while the combined model remains strongest, indicating complementary sequence and topology signals. This indicates that *positional information provides a measurable benefit*.

### 3.2 Benchmarking against state-of-the-art methods

We benchmark RIBEX against two classes of state-of-the-art RBP prediction methods. First, *RBP-TSTL* (Peng *et al*. 2022), which to our knowledge represents the current state of the art in RBP prediction using pLM representations, also shown to outperform earlier non–deep learning approaches such as TriPepSVM. Second, we compare against network-based and hybrid approaches, including *SONAR 3.0* (Brannan *et al*. 2016), which predicts RBPs by exploiting the observation that proteins interacting with multiple RBPs are themselves likely to bind RNA, and HydRA (Jin *et al*. 2023), a hybrid deep-learning framework that integrates protein sequence features with PPI network information.

RIBEX with ProtT5-XL embeddings and PEs already significantly outperforms the baseline RBP-TSTL configuration even without LoRA fine-tuning, showing relative AUPRC improvements of 7.5% and 10.8% on the Bressin and RIC benchmarks, respectively (Fig. 2C, D), and corresponding relative MCC gains of 7.1% and 9.3% (Supplemental Fig. 6C, D, available as supplementary data at *Bioinformatics* online). Replacing ProtT5-XL with ESM2-650M slightly improves the RBP-TSTL baseline, particularly on the Bressin dataset. However, the RIBEX architecture consistently outperforms both configurations across all benchmarks, with significant AUPRC improvements (Fig. 2) and corresponding MCC gains (Supplemental Fig. 6C, D, available as supplementary data at *Bioinformatics* online). Motivated by the RBP-TSTL design, we also tested multi-species pretraining in our pipeline using *E. coli*, *Salmonella*, *Mus musculus*, and *Saccharomyces cerevisiae*; however, this strategy did not improve performance in our setting (see Supplemental Section B.2, available as supplementary data at *Bioinformatics* online).

*On the HydRA benchmark* (Jin *et al*. 2023), we run RIBEX on the same dataset and compare against the published test set predictions distributed with HydRA, including HydRA-seq (the sequence-only variant with ablated network information) and SONAR 3.0 (Brannan *et al*. 2016). Details of the evaluation protocol and metrics are provided in Supplemental Section A.2, available as supplementary data at *Bioinformatics* online, with additional plots in Supplemental Section B.3, available as supplementary data at *Bioinformatics* online. In these supplementary comparisons, SONAR 3.0 performs below both HydRA and the evaluated RIBEX variants, supporting that PPI context is informative but benefits from integration with sequence information (Supplemental Fig. 8, available as supplementary data at *Bioinformatics* online). Panels G and H of Fig. 2 report AUPRC together with TopK-AUC, defined as the area under the Precision-at-top-*K* curve for k≤150. In this setting, both RIBEX variants—ESM2-650M with LoRA and ProtT5-XL without LoRA—outperform the HydRA baseline. For AUPRC, relative improvements over HydRA are approximately 5.9% and 6.6% on the subset lacking classical RBDs for ESM2-650M and ProtT5-XL, respectively, and approximately 2.5% and 2.7% on the classical-RBD subset (Fig. 2G, H). Notably, for proteins lacking classic RNA-binding domains (panel G) the relative drop in performance is smaller for RIBEX than for HydRA when compared with the classic-RBD subset (panel H), indicating improved robustness on this harder subset.

### 3.3 Explainability

To probe which aspects of the model’s learned representation drive its predictions, we apply two complementary perturbation-based analyses detailed in Section 2.5: a network-level positional encoding scan (Fig. 3A) and a sequence-level computational alanine scan (Fig. 3B).

#### 3.3.1 Positional encoding scan identifies functional interactome communities

Applying the network-level feature ablation strategy (Section 2.5) to high-confidence true-positive RBPs can in principle identify relevant partners for individual proteins, but these single-protein explanations remain noisy. We therefore aggregated the most affected neighbour nodes across top predictions, yielding a per-node frequency score that highlights network regions consistently used by the model. When overlaid on a t-SNE embedding of the PE matrix (Fig. 3A), these recurrent neighbour nodes form spatially coherent clusters. We delineated these regions with DBSCAN and performed GO biological-process enrichment, revealing processes where RBPs play key roles, including cytoplasmic translation, ribosome biogenesis, cytoskeleton organization, ER stress-related localized translation, and centrosome/cell-cycle functions. As one example, the ribosome-biogenesis-related cluster contains known ribonucleoprotein-complex components such as IMP3, SNU13, and NHP2, supporting that the model captures functionally coherent interactome regions and, in some cases, known RNA-associated protein complexes (Watkins *et al*. 2000; Granneman *et al*. 2003; Wang and Meier 2004); see Supplemental Section A.4.1, available as supplementary data at *Bioinformatics* online and Fig. 4.

#### 3.3.2 Alanine scanning resolves domain and IDR contributions

To complement the network-level analysis, we applied *in silico* alanine scanning (Section 2.5) to correctly predicted RIC-positive test proteins. The resulting per-window probability changes ($\Delta {\hat{y}}_{\text{Ala}}$), where strong negative peaks indicate important sequence segments, are visualized together with smoothed AlphaFold confidence scores and domain/IDR annotations (Fig. 3B).

We observed that more negative scores—corresponding to regions predicted to be more important for RNA binding—tended to occur closer to protein–RNA interfaces, where interface residues were defined as those within 10 Å of any RNA atom (Supplemental Fig. 11, available as supplementary data at *Bioinformatics* online). This enrichment supports the ability of the alanine scan to identify functionally relevant RNA-binding regions.

The revised case studies recover established RNA-binding biology across three complementary examples. In PUM2, the strongest sensitivity overlaps the C-terminal PUM-HD domain, the canonical sequence-specific RNA-binding module, with additional signal in the N-terminal disordered region overlapping the RD2 segment (Lu and Tanaka Hall 2011). In TARDBP/TDP-43, coherent negative signal spans RRM1 and RRM2, consistent with the tandem RRM architecture responsible for UG-rich RNA recognition (Lukavsky *et al*. 2013). In LIN28A, the alanine scanning highlights three important regions: the two canonical RNA-binding domains—the Cold-shock domain (CSD) and the CCHC zinc-knuckle domain, which are known to act cooperatively to confer high-affinity RNA binding, and the N-terminal intrinsically disordered region, which further enhances binding through cooperative interactions with the structured domains (Nam *et al*. 2011; Mayr *et al*. 2012). Additional examples, especially IDR-rich RBPs with low-complexity, basic, or RG/RGG-rich RNA-associated regions such as SERBP1, TIA1, DDX4, and FUS, are discussed in Supplemental Section C.3 and Fig. 12, available as supplementary data at *Bioinformatics* online, with broader alanine-scan profiles provided in Supplemental Fig. 13, 17 and 18, available as supplementary data at *Bioinformatics* online.

## 4 Discussion

Here we present RIBEX as a protein-level framework for prioritizing canonical and non-canonical RBPs by integrating sequence information with network topology; its output is intended to represent direct and indirect RNA-binding propensity, not residue-level evidence of direct RNA contact. Across benchmarks, positional encodings (PE) consistently improve performance, supporting our central premise that network topology provides information complementary to sequence features. Accordingly, RIBEX remains competitive with or outperforms strong baselines such as RBP-TSTL and HydRA (Fig. 2C, D, G, H), despite using a comparatively simple FiLM-based fusion mechanism. A supplementary centrality analysis further indicates that predicted RBPs are not shifted toward more highly connected nodes than labeled RBPs, although true RBPs show the expected higher centrality (Brannan *et al*. 2016) than true non-RBPs (Supplementary Figs. 9 and 10, available as supplementary data at *Bioinformatics* online).

*Ablation results* show that task-specific LoRA adaptation on ESM2-650M improves performance more than simply scaling the frozen pLM backbone (Fig. 2E, F). The limited effect of backbone size, especially on the challenging RIC benchmark, indicates that human-only RBP prediction depends not just on sequence representation but also on task adaptation and contextual information, consistent with the additional gains from PE after LoRA fine-tuning. *Dataset differences* are informative: the Bressin dataset is annotation-based, enriched for canonical RBPs, while the RIC dataset captures experimentally detected RNA associations, including indirect or context-dependent interactions. Methodologically, RIBEX differs from HydRA in how sequence and network evidence are represented and fused: HydRA combines separately trained sequence and SONAR 3.0 interaction classifiers by late probabilistic ensembling, whereas RIBEX FiLM-conditions a LoRA-adapted pLM on PPR-based STRING positional encodings during the forward pass. This coupled fusion of a task-adapted sequence model with broader graph-topology features may explain the stronger topology signal and the smaller performance drop on non-canonical RBPs (Supplemental Section A.2.1, available as supplementary data at *Bioinformatics* online). The *interpretability analyses* further support this view.

At the sequence level, computational alanine scanning highlights sensitivity in known RNA-binding domains, as well as selected IDR segments. These attributions should be interpreted as model-sensitivity maps for protein-level RNA-binding or RNA-associated propensity: they cannot by themselves distinguish residues that directly contact RNA from regions that support RNA-associated behaviour indirectly, for example through protein–protein interfaces required for RNA-binding complex assembly. Thus, examples such as PUM2, TARDBP/TDP-43, and LIN28A support recovery of known RNA-recognition biology, whereas AFF4 is discussed more cautiously as RNA-associated or complex-context evidence (Supplemental Section C.3, available as supplementary data at *Bioinformatics* online). At the network level, PE ablation identifies influential interactome neighbourhoods rather than causal binding mechanisms; the highlighted IMP3, SNU13/NHP2L1, and NHP2 examples illustrate RNP-complex context (Supplemental Section A.4.1, available as supplementary data at *Bioinformatics* online).

Accordingly, the main limitations are that RIBEX predicts protein-level RNA-binding/RNA-association propensity without resolving RNA targets or residue-level contacts, uses a single STRING-based human interactome, and provides high-level influential neighbourhoods rather than causal subgraph explanations. Alanine-scan peaks should therefore be read as candidate domains, IDR/interface regions, or RNA-complex determinants contributing to the prediction, not as definitive RNA-contact residues.

Future improvements could include tighter sequence–graph integration via pLM-based node features or cross-modal message passing, alternative PEs, and extensions to cross-species or protein–RNA interaction prediction, all aimed at boosting predictive accuracy and supporting more interpretable, subgraph-level reasoning.

Taken together, our results position RIBEX as a practical framework for prioritizing candidate RBPs and generating mechanistic hypotheses, with complementary interpretability particularly valuable for non-canonical proteins where sequence alone is insufficient.

## Supplementary Material

btag471_Supplementary_Data

## Data Availability

The RIBEX codebase, including data-regeneration scripts, is available under MIT license at GitHub (URL) and archived at the Software Heritage Archive (URL).
