# Supplementary material for: RIBEX: predicting and explaining RNA binding across structured and intrinsically disordered regions (IDR)-rich proteins
Source: Bioinformatics. 2026 Aug 21;42(Suppl 2):btag471. doi: 10.1093/bioinformatics/btag471 (PMC13501326; doi:10.1093/bioinformatics/btag471)
Supplement: Supplementary file 1 [file btag471_supplementary_data.pdf]

Supplementary Material

RIBEX: Predicting and Explaining RNA Binding Across Structured and Intrinsically Disordered Regions (IDR)-rich Proteins

Samuele Firmani, Felix Steinbauer, Gjergji Kasneci, Annalisa Marsico†, Marc Horlacher†

†These authors contributed equally.

A. Methods and Experimental Details

A.1. Dataset Details

For reproducibility, transparency, and discussion of potential limitations, we describe the original datasets used in more detail.

A.1.1. Original Datasets

As the same preprocessing is applied to all datasets, we here describe each source dataset individually.

Bressin dataset from (Bressin et al. 2019)

As mentioned in Section 2, the Bressin dataset uses the UniprotKB database (Consortium 2025) and flags RNA-*binding-positive* proteins using the QuickGO (Binns et al. 2009) term "GO:0003723". This flag indicates only **known** binding-positive behavior, but determining *binding-negative* proteins requires a more involved approach: the authors first collect all human proteins from Swiss-Prot and remove those with sequence lengths below 50 AA or above 6000 AA. They then gather UniProt keywords for each protein and exclude those matching a list of binding-associated keywords. Proteins for which QuickGO reports a DNA-, RNA-, or nucleotide-binding flag are likewise excluded. Finally, Pfam (Bateman et al. 2004) domains associated with RNA binding serve as additional exclusion criteria. All remaining proteins make up the *binding-negative* class. For more details, see (Bressin et al. 2019) and the respective Supplementary Material.

For all sequences, CD-Hit (Li and Godzik 2006) is applied to remove proteins with sequence similarity above 90%. The final human dataset contains 1812 binding-positive and 12038 binding-negative proteins.

RIC dataset from (Hentze et al. 2018)

The RIC dataset (available from (RBP 2026)) focuses on RNA-binding behavior and contains RNA interactome capture (RIC)-based experimental results for most proteins. It reports whether a protein was RNA-binding positive across multiple cell lines. In this paper, we consider the cell-based RIC assays for the human cell lines HeLa, JURKAT, MCF7, and HEK293. More precisely, we consider the following 8: `Hs_HeLa-RIC` (`RBPBASE000000008.1`), `Hs_HeLa-RBDmap` (`RBPBASE000000009.1`), `Hs_JURKAT-2018-eRIC` (`RBPBASE000000034.1`), `Hs_HeLa-RICK-hc` (`RBPBASE000000035.1`), `Hs_XRNAX-HeLa` (`RBPBASE000000059.1`), `Hs_XRNAX-HEK293` (`RBPBASE000000060.1`), `Hs_XRNAX-MCF7-ars` (`RBPBASE000000061.1`), `Hs_HEK293-cRIC-SINV` (`RBPBASE000000062.1`). As some proteins bind RNA in only one or a few cell lines while others do so in many or all, the RNA-binding property is not strictly binary in this dataset. To define *binding-positive* and *binding-negative* classes for model training, we classify as binding negative all proteins showing no RNA binding in any of the above-mentioned cell lines, and as binding positive all others (i.e., binding exhibited in at least one of the 8 cell lines). Thus, no additional random negative sampling is introduced for the RIC dataset; the negative class is defined directly from the absence of RNA-binding evidence across the selected assays. We acknowledge that this binarisation simplifies what is likely a continuous RNA-binding affinity spectrum.

HydRA dataset from (Jin et al. 2023)

The dataset used for HydRA (Jin et al. 2023) (available on their GitHub (URL)) comprises annotated protein sequences and protein–protein interaction (PPI) network metadata. For their PPI network, they combine BioPlex2.0 (Huttlin et al. 2015) with reliability scores derived from experimental data in the MINT (Licata et al. 2012) and Mentha (Calderone et al. 2013) databases, and supplement these with functional associations from the STRING database (Szklarczyk et al. 2023).

The protein sequences for the proteins in their PPI network are retrieved from UniProt (Consortium 2025), and RBP-positive flags are collected from diverse sources including QuickGO (Binns et al. 2009) and AmiGO2 (Ashburner et al. 2000, The Gene Ontology Consortium 2026) terms. Based on these annotations, Jin et al. (2023) define the *binding-positive* and *binding-negative* classes; the original HydRA resource comprises 18751[1] proteins, of which 3762 (19.85%) are positive. For our comparison, we did not retrain HydRA: we used the published HydRA labels, held-out split, and prediction scores, while mapping HydRA Table S2 `Gene_IDs` to our curated sequence table to generate the corresponding RIBEX inputs. This mapping recovered 16858 of 18751 Table S2 entries (∼90%); after removing 251 entries with missing or empty sequences, the final RIBEX input set for the HydRA comparison contained 16607 proteins.

HydRA dataset variation: non-RBD sequences

Canonical RNA-binding domains (RBDs) such as RRM or KH domains are strong, easily detectable sequence signals; proteins carrying them are straightforwardly classified as RBPs by almost any sequence-based method. To probe each model's capacity to detect RNA binding in the absence of such canonical features—which is particularly relevant for IDR-mediated RNA association—we exclude sequences with known RBDs from the test set. Positive proteins are retained only if they are annotated as RBPs in the curated catalogue (Gerstberger et al. 2014) *and* carry no annotated RBD (i.e., zero known RNA-binding domains, zero RBD sequence coverage). All negative proteins are kept without

[1] Their paper reports 18951, but the dataset contains 18751 entries.

modification. Easy positives—those whose RNA-binding activity is readily predictable from canonical domain content—are thereby excluded from the evaluation.

A.1.2. Homology Clustering and Leakage Control

For each source dataset used for RIBEX training or evaluation, protein sequences were exported from the curated TSV tables and clustered with MMseqs2 (Steinegger and Söding 2017) using `--min-seq-id 0.2 -c 0.5 --cov-mode 0`. With MMseqs2 `--cov-mode 0`, the coverage criterion is computed relative to the longer of the two aligned sequences; therefore, clustered proteins share at least 20% sequence identity over at least 50% of the longer protein. The resulting assignments were stored as `cluster_number`. During split generation, `cluster_number` was used as the grouping variable. Proteins without assigned neighbours were treated as singleton groups. Group-based splitting ensures that all proteins from the same MMseqs2 cluster are assigned to the same partition, preventing homologous proteins from appearing simultaneously in training and validation/test subsets. This follows the motivation of previous RBP benchmarks while using a stricter sequence-identity cutoff. (Bressin et al. 2019) used CD-HIT (Li and Godzik 2006) to remove proteins with sequence similarity above 90%, mainly to avoid duplicate annotations or paralogs in UniProtKB. (Peng et al. 2022) used CD-HIT at a 25% identity threshold to reduce redundancy among training, validation, and independent test datasets. Our MMseqs2 threshold of 20% identity, combined with cluster-level splitting, is therefore at least as stringent as these prior controls.

A.1.3. Splitting into Training, Validation, and Test Sets

Tables 1–2 report the size and class composition of the training, validation, and test partitions used for benchmarking. Reported values correspond to a single representative seed (seed 12345); partition sizes are identical across all ten seeds by construction, as the common gene pool is fixed.

Table 1 Split statistics for the (Hentze et al. 2018)-based dataset (RIC) and the (Bressin et al. 2019)-based dataset (Bressin). The common gene pool comprises 15,299 proteins for the RIC dataset (**left**) and 10,414 proteins for the Bressin dataset (**right**). RNA-binding positives constitute roughly 9–14% of each pool, reflecting substantial class imbalance. Only proteins for which embeddings are available for **all** four evaluated pLMs are retained. For both datasets, 10% of proteins form the hold-out set, split into a validation fold (one third; used for epoch selection) and a test fold (two thirds; used for final evaluation).

	(Hentze et al. 2018)-based (RIC)		(Bressin et al. 2019)-based (Bressin)	
**Split**	**Samples**	**RBP-positive**	**Samples**	**RBP-positive**
Train	13,769	1,430 (10.4%)	9,372	1,208 (12.9%)
Validation	510	48 (9.4%)	347	47 (13.5%)
Test	1,020	96 (9.4%)	695	93 (13.4%)
Total	*15,299*	*1,574 (10.3%)*	*10,414*	*1,348 (12.9%)*

Table 2 Split statistics for the (Jin et al. 2023)-based dataset (HydRA), stratified by pLM. The training set comprises all proteins from the original dataset not included in the original test set (see (Jin et al. 2023)). Training set sizes vary across pLMs because only proteins for which embeddings are available are retained; the test set is taken directly from the HydRA repository and is identical in size across all models. Per-seed validation proteins for epoch selection are derived by bootstrap resampling with replacement from the test set (out-of-bag samples) and are not listed as a separate partition.

Model	Train	RBP-positive	Test	RBP-positive
ProtT5-XL	12,974	2,588 (19.9%)	2,994	575 (19.2%)
ESM2 650M	12,645	2,459 (19.4%)	2,994	575 (19.2%)
ESM2 3B	12,381	2,358 (19.0%)	2,994	575 (19.2%)

A.2. Benchmark Evaluation Protocol

All comparisons against HydRA (Jin et al. 2023), HydRA-seqOnly, and SONAR 3.0 (Brannan et al. 2016) are performed on the HydRA held-out test set. For each independent random seed, we restrict all models to their common protein coverage by computing the intersection of proteins available in every compared model. For each seed, metrics are computed on a bootstrap sample drawn with replacement from this shared held-out test pool. For each RIBEX variant, the training checkpoint that maximises AUPRC on the validation subset is selected; predictions from this checkpoint are then generated on the held-out test subset. Baseline scores for HydRA, HydRA-seqOnly, and SONAR 3.0 are fixed post-hoc predictions and are evaluated unchanged on the same bootstrap-sampled test proteins.

Evaluation metrics.

Two retrieval metrics are computed on the filtered test set. The **area under the precision-recall curve (AUPRC)** summarises overall discriminative performance under class imbalance; because positives are a small minority of the human proteome, AUPRC is more informative than Area Under the Receiver Operating Characteristic Curve (AUROC) in this setting. **Precision at top** K (Precision at Top K (P@K)) measures the fraction of true RBPs among the K highest-scoring proteins for $K \in [10, 300]$, directly quantifying the practical utility of each model for prioritising experimental follow-up. The area under the P@K curve (TopK-AUC) is computed by numerical integration of P@K over K after linearly rescaling K to $[0, 1]$, so that the score summarizes ranking quality across the selected cutoff range. In the main-text HydRA comparison (Figure 2G,H), we report the restricted variant computed over $K \leq 150$ to emphasize the regime most relevant for actionable candidate lists.

Statistical testing.

Comparisons are performed over twelve independent random seeds. Seed-matched metric pairs (i.e., values from the same partition) are compared with a two-sided paired t-test. When multiple models are tested against HydRA simultaneously, p-values are adjusted with the Holm–Bonferroni procedure (Holm 1979).

A.2.1. Methodological comparison with HydRA

HydRA is a hybrid ensemble classifier that combines an interaction-context component, SONAR 3.0, with three sequence-based classifiers: seqSVM, seqCNN, and ProteinBERT-RBP. These components are trained as separate predictors and combined through a false-discovery-rate-based probabilistic ensemble (Jin et al. 2023). Thus, sequence and network evidence are primarily fused at the score level. RIBEX instead uses a single multimodal architecture. The sequence branch is a LoRA-adapted ESM-2 pLM (Hu et al. 2021, Lin et al. 2022), while network context is represented by Personalized PageRank vectors over the STRING PPI graph (Page et al. 1999, Szklarczyk et al. 2023), reduced by PCA. A FiLM layer (Perez et al. 2018) uses these graph-derived positional encodings to scale and shift the pooled sequence representation before classification, so the network context conditions how sequence evidence is interpreted during the forward pass rather than being combined only after independent predictions. The network summaries also differ in scope. HydRA's SONAR3.0 branch represents a protein through hand-engineered local PPI-neighbourhood summaries, including how many nearby proteins are known RBPs and whether the node is sparsely connected. RIBEX's PPR-based positional encodings instead capture broader random-walk proximity and global topological roles in the interactome. This design supports the modality ablations in Figure 2A,B and the inverse-PCA PE-ablation explanation strategy in Figure 3A and Suppl. Figure 4.

A.3. Hyperparameters

Tables 3 and 4 report the final hyperparameters used for all RIBEX model variants. For the RIC and Bressin dataset, hyperparameters were held fixed; for the HydRA benchmark, LoRA hyperparameters were determined by random search and are model-specific. FiLM PE settings are identical across all four pLMs within each benchmark. All LoRA adapters target the key and value projection matrices of the self-attention layers. The controlled comparison between LoRA adaptation and backbone scaling in Figure 2E,F is therefore restricted to LoRA on ESM2 650M versus larger frozen backbones. We also ran preliminary LoRA experiments on larger ESM2 backbones, but these runs did not improve over the corresponding frozen models and were limited by substantially smaller feasible batch sizes and incomplete hyperparameter tuning; consequently, they are not used to support a general claim that LoRA always outperforms backbone scaling. The LoRA settings reported in Table 4 correspond to the model variants retained in the final benchmark.

Table 3 FiLM PE hyperparameters. Settings are shared across all pLMs (ESM2 650M, ESM2 3B, ESM2 15B, ProtT5-XL) within each benchmark. d_{PE}: number of PCA components retained for the positional encoding. Patience refers to the early-stopping patience in epochs (monitored on validation AUPRC).

Benchmark	Learning rate	Batch size	$d_{\mathbf{PE}}$
RIC / Bressin	5.0×10^{-4}	256	512
HydRA	9.3×10^{-5}	256	512

Table 4 LoRA hyperparameters. For RIC and the Bressin dataset, the same settings are used for both ESM2 650M and ProtT5-XL; rank, α, and dropout were left at default values in those benchmarks. For HydRA, hyperparameters were optimised separately per backbone via random search. r: LoRA rank; α: LoRA scaling factor; WD: weight decay; d_{PE}: PCA components for positional encoding. Patience refers to early-stopping patience (validation AUPRC).

Benchmark	Model	LR	BS	$d_{\mathbf{PE}}$	r	α	Dropout	Weight Decay
RIC / Bressin	ESM2 650M	3.0×10^{-4}	256	512	3	0.42	0.45	5.3×10^{-4}
	ProtT5-XL	3.0×10^{-4}	256	512	3	0.42	0.45	5.3×10^{-4}
HydRA	ESM2 650M	9.1×10^{-4}	415	418	6	1.66	0.38	3.7×10^{-4}
	ProtT5-XL	3.4×10^{-4}	392	466	10	1.58	0.38	1.6×10^{-4}

A.4. Positional Encoding Experiments

Figure 4 shows the aggregated node-frequency map from the inverse-PCA PE-scan analysis, overlaid on the t-SNE embedding of the full PE matrix. The literature-supported partner examples highlighted in the figure are discussed in Section A.4.1. Figure 5 provides a complementary channel-level t-SNE view of the PE space, shown separately for the Bressin and RIC datasets.

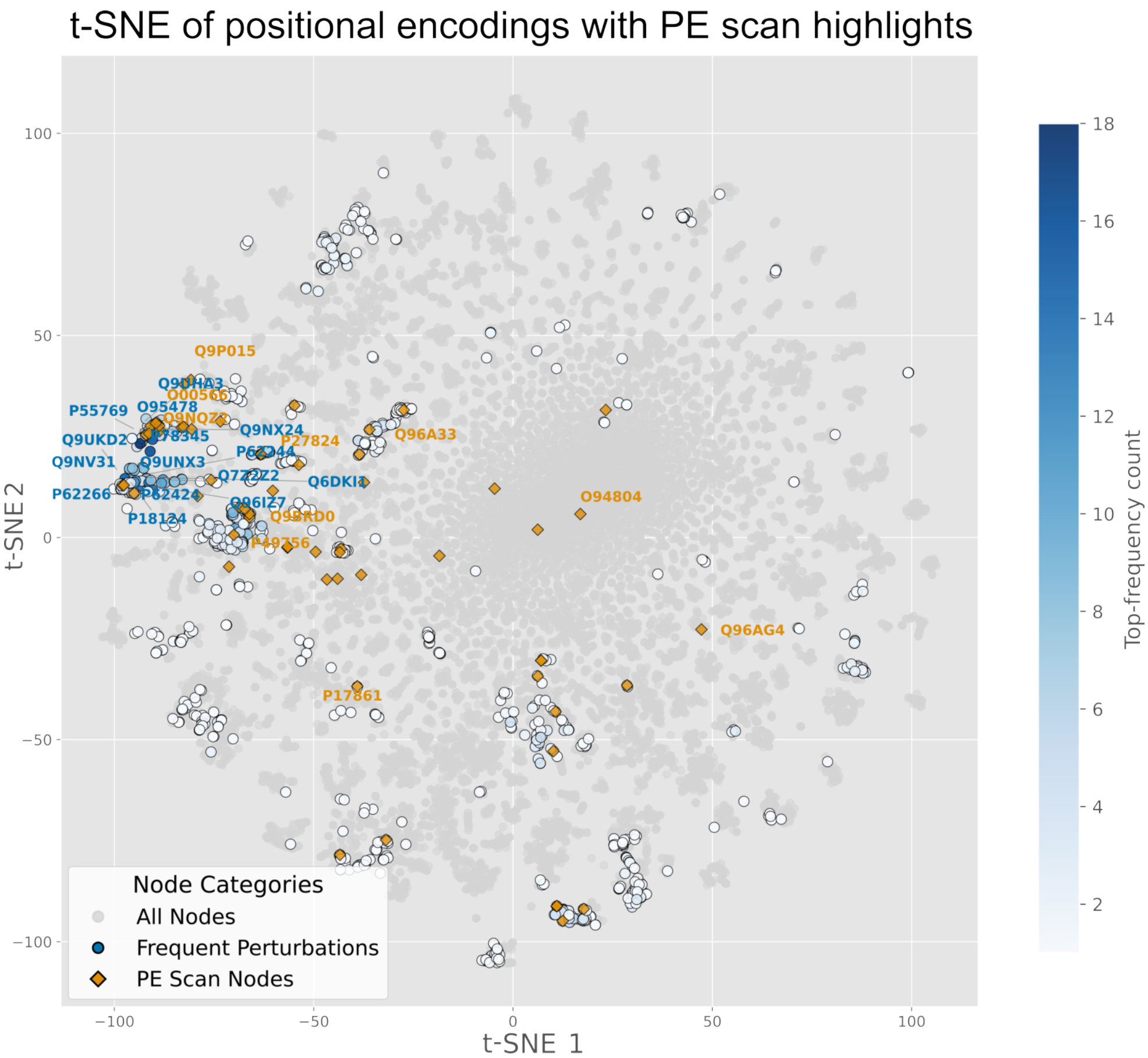

Figure 4 Frequency map of the positional-encoding explanation analysis. Nodes are shown in the reduced PE t-SNE space together with the aggregated frequency counts from the inverse-PCA PE-scan analysis. Blue points denote interacting partners, and orange points denote the top predicted positives in the held-out test set. Blue labels mark additional model-derived partner nodes, while orange-highlighted partner labels mark the literature-supported RNP case studies discussed below.

A.4.1. Literature-supported PE partner examples

To assess whether the model-derived partner nodes reflected known RNP biology, we focused on three literature-supported examples from the explanation map: `Q9NV31`, `P55769`, and `Q9NX24`. These examples are intended to illustrate RNP-complex context: ribonucleoprotein complexes are assemblies in which RNA is bound, folded, processed, or stabilized together with multiple proteins, so a model-derived partner can be biologically meaningful when it identifies a protein that binds RNA directly or helps nucleate such RNA-bound assemblies. `Q9NV31` encodes *IMP3*, a U3 snoRNP protein shown to form a ternary complex with *IMP4* and hMpp10/MPHOSPH10 in human 60–80S U3-containing ribonucleoprotein complexes (Granneman et al. 2003). hMpp10/MPHOSPH10 corresponds to `O00566`, one of the labelled orange RBP nodes in the explanation map, linking this partner neighbourhood to the U3 snoRNP module. `P55769` encodes SNU13/NHP2L1, the 15.5K protein, a k-turn RNA-binding factor that contributes to 15.5K-mediated assembly of U4/U6 snRNPs and box C/D snoRNPs (Watkins et al. 2000, Schultz et al. 2006). `Q9NX24` encodes NHP2, a conserved core H/ACA RNP protein that assembles with dyskerin/DKC1, NOP10, and GAR1 in mammalian H/ACA snoRNP and telomerase RNP particles (Wang and Meier 2004). Thus, the highlighted blue nodes are not interpreted as isolated annotations, but as model-derived partners that recover known RNA-bound protein-complex neighbourhoods.

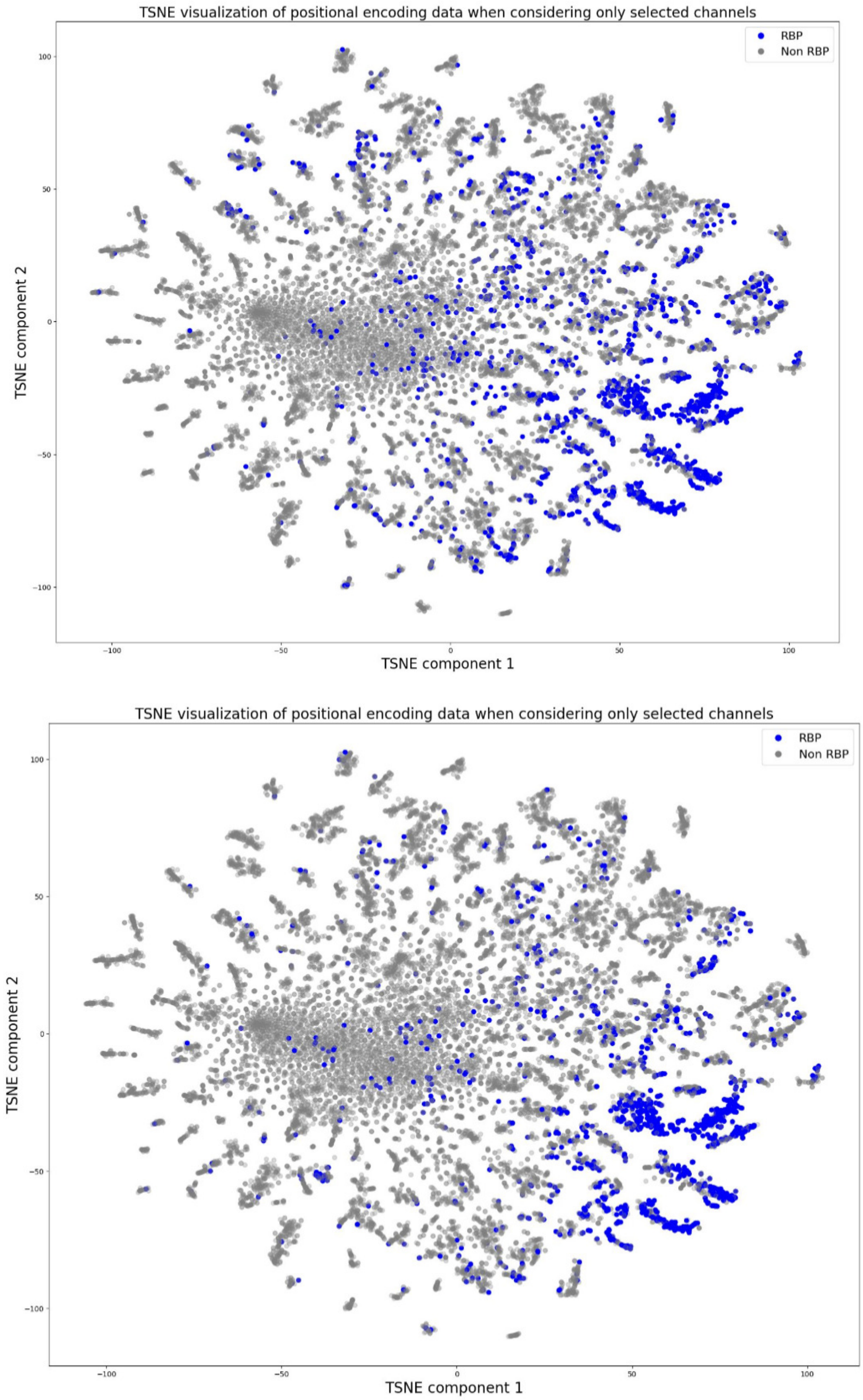

Figure 5 t-SNE Visualization of Positional Encoding Data for Selected Channels. Top: Bressin dataset positional-encoding subset. **Bottom:** RIC positional-encoding subset.

B. Additional Results

B.1. Additional Benchmark Figures

The main benchmark figure (Figure 2) reports AUPRC as the primary metric for each experimental condition. Figure 6 extends this comparison with the full evaluation metric set—additionally including MCC—across all conditions and both the Bressin and RIC datasets.

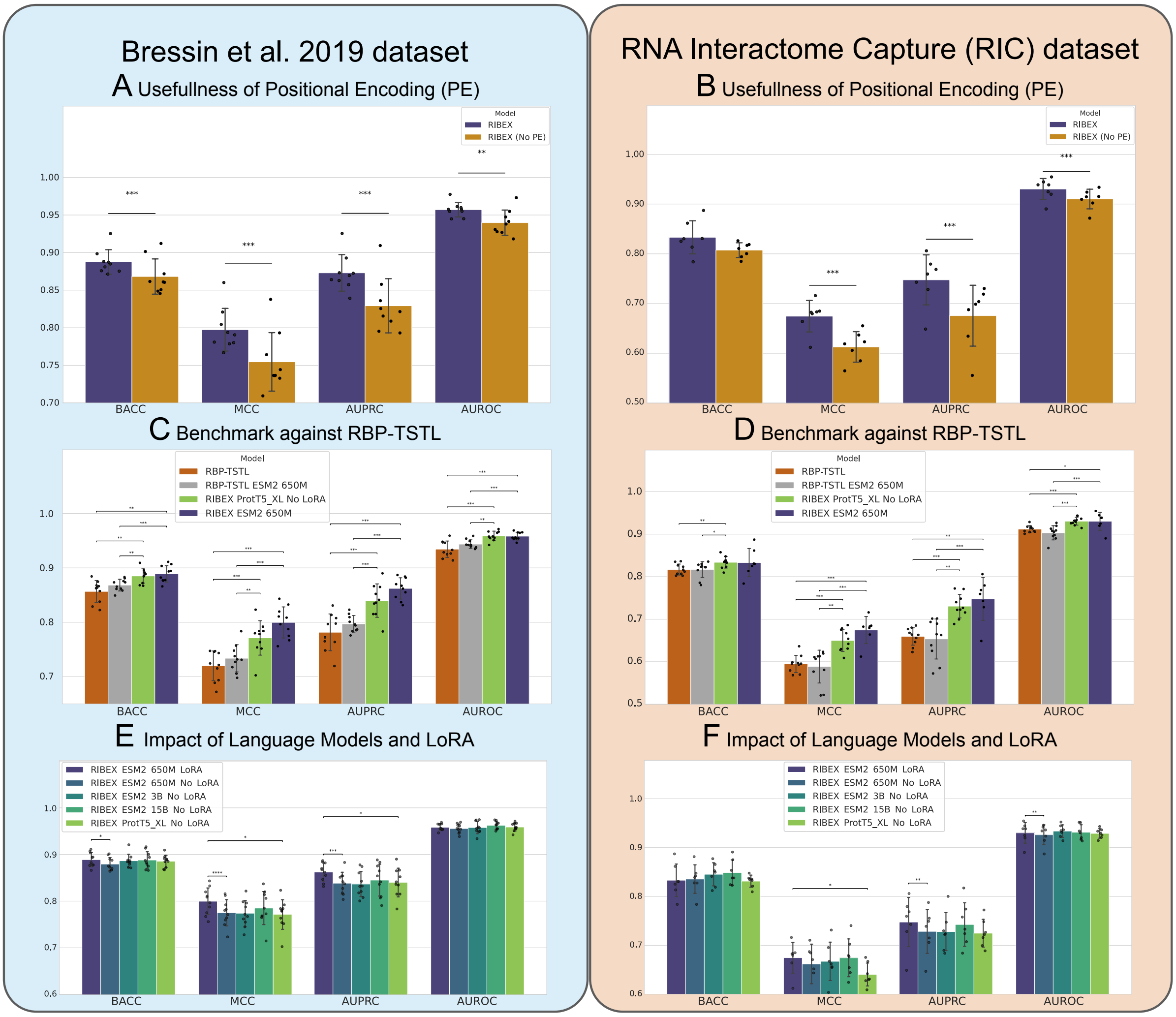

Figure 6 Full benchmark comparison across all reported metrics. Panels **A–B** show the effect of positional encodings on the Bressin dataset and RIC benchmarks, respectively. Panels **C–D** compare RIBEX against RBP-TSTL on the Bressin dataset and RIC. Panels **E–F** show the effect of pLM backbone choice and LoRA fine-tuning on the RIC and Bressin dataset. This expanded supplementary figure includes the full metric set used during benchmarking and is therefore referenced in the main text for metrics not shown in Figure 2.

B.2. Pretraining Experiments

Motivated by the multi-species pretraining strategy of RBP-TSTL (Peng et al. 2022), we evaluated whether pretraining RIBEX on non-human species (*E. coli*, *Salmonella*, *Mus musculus*, and *Saccharomyces cerevisiae*) before fine-tuning on human data improves performance. Figure 7 shows that pretraining did not yield consistent improvements in our human-only setting and was therefore not adopted in the main benchmarks.

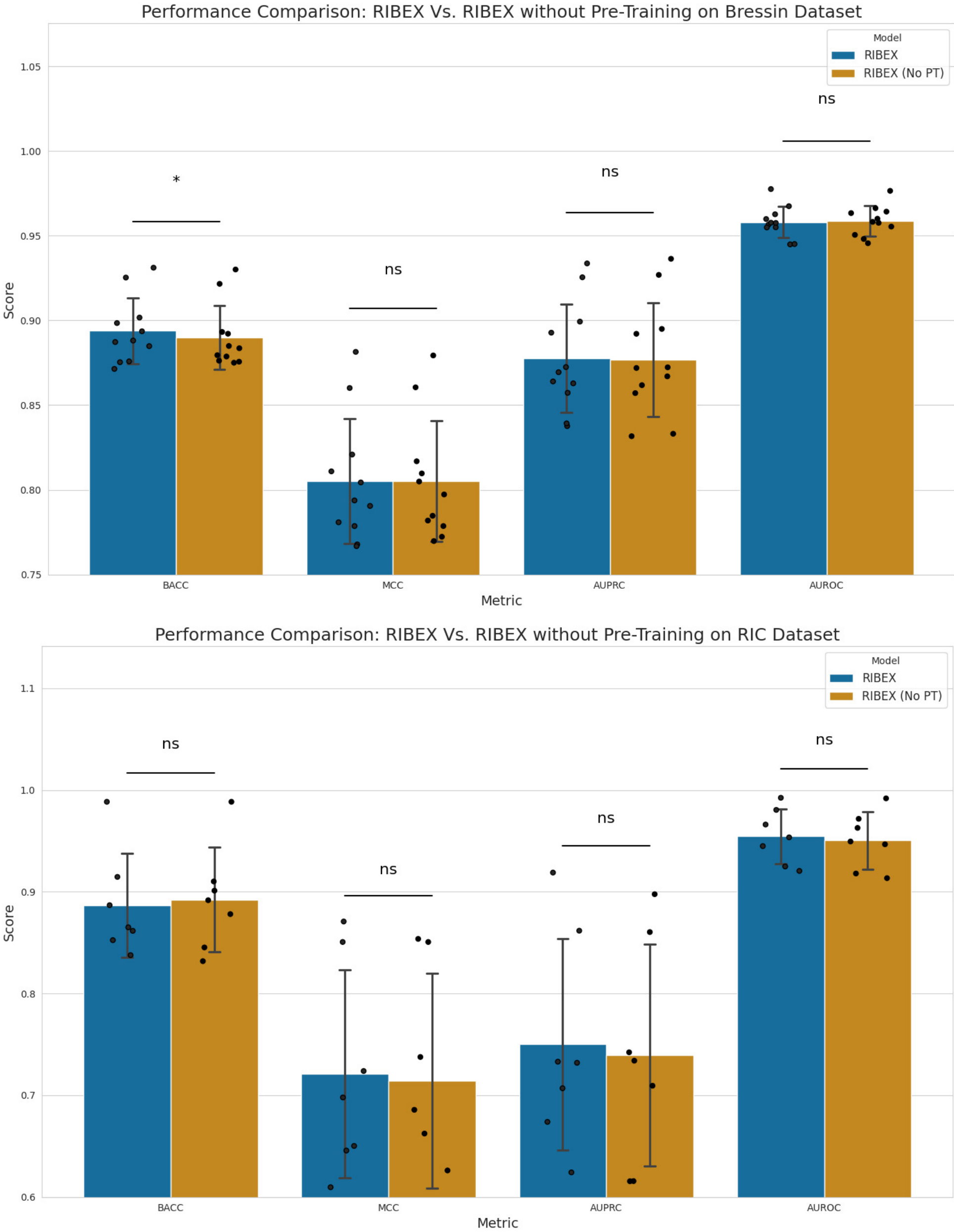

Figure 7 Performance Comparison Between RIBEX and RIBEX Without Pre-Training. Top: Bressin dataset **Bottom:** RIC dataset

B.3. HydRA Supplementary Figures

Figure 8 provides the complete pairwise comparison between RIBEX variants, HydRA (Jin et al. 2023), HydRA-Sequence-Only, and SONAR 3.0 (Brannan et al. 2016). Both AUPRC and Precision-at-top-K are reported for all test-set positives (top) and for the harder subset restricted to proteins without canonical RBDs (bottom), complementing the summary panels in Figure 2G,H.

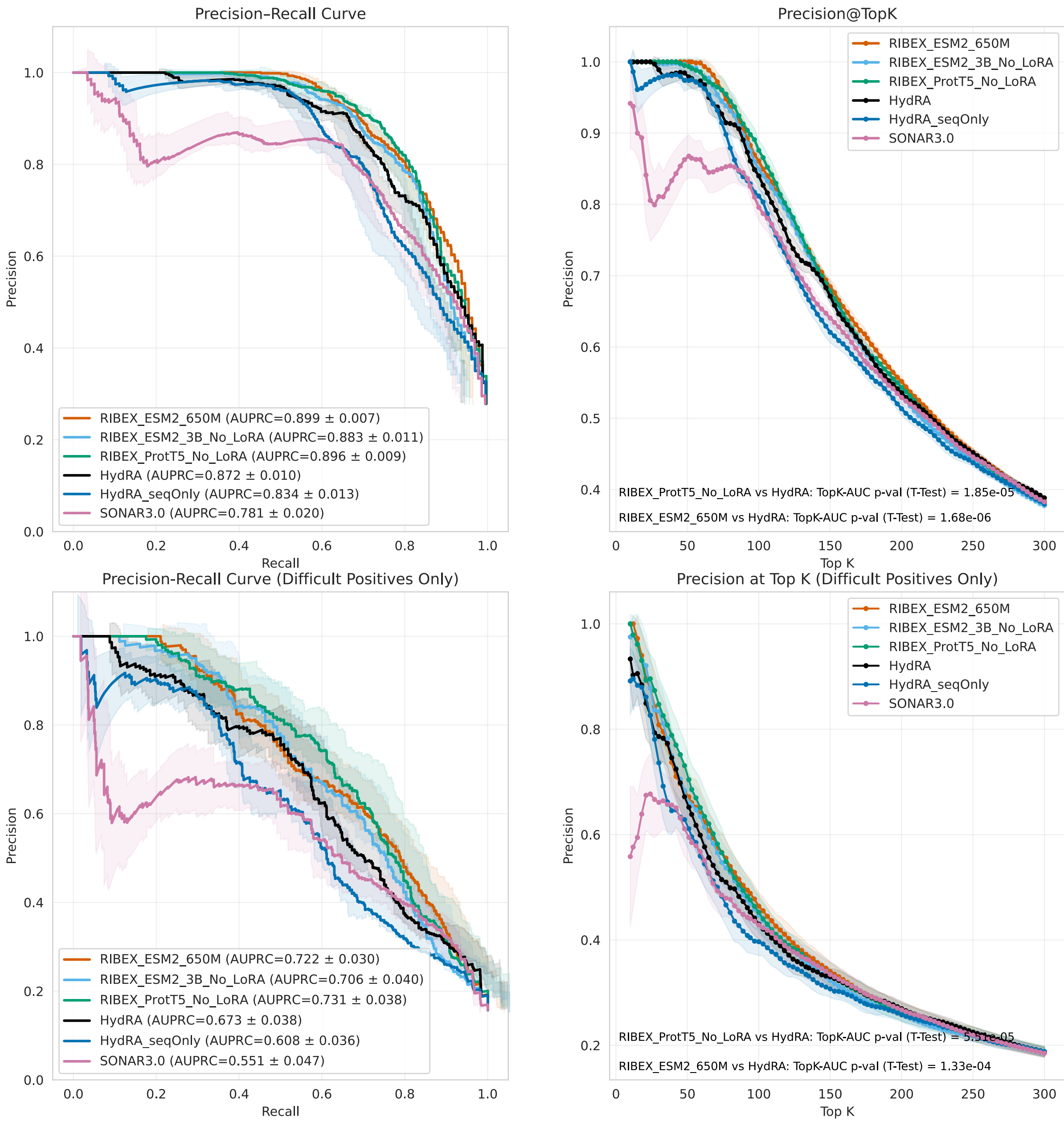

Figure 8 AUPRC and Precision-at-Top-K Comparison Between HydRA (Jin et al. 2023), HydRA-Sequence-Only, SONAR 3.0 (Brannan et al. 2016), and RIBEX. Using a paired t-test to compare against RIBEX (ESM2, 650M), RIBEX (ESM2, 3B) without LoRA, and RIBEX (ProtT5) without LoRA. Comparison is done on all test-set positives (**Top**) and on only those not showing any canonical RNA-binding domain (**Bottom**).

B.4. Prediction Centrality Comparisons

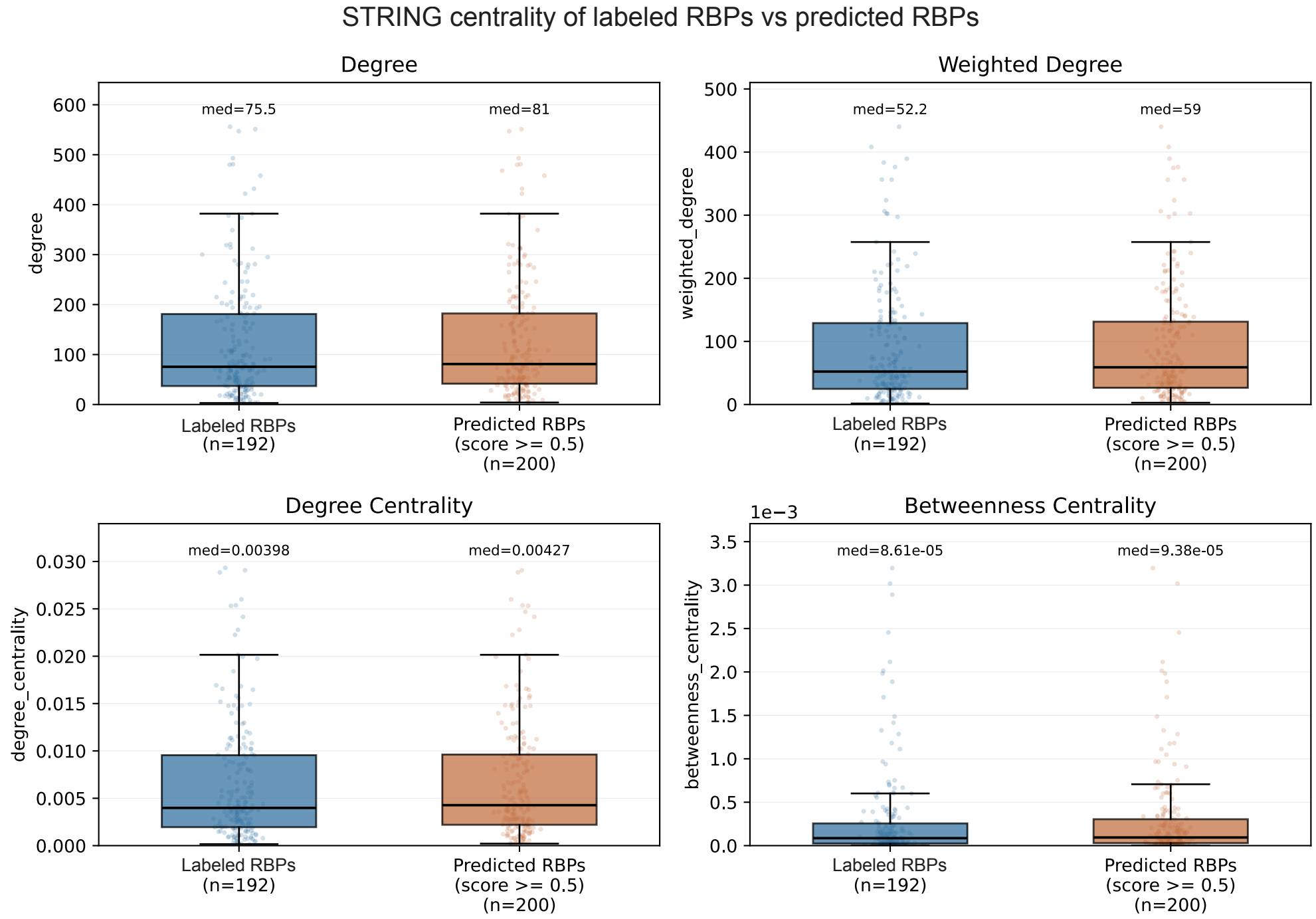

Figure 9 Centrality comparison of labeled vs. predicted RBPs. Network centrality measures are compared between proteins annotated as RNA-binding proteins (labeled RBPs) and those predicted as RNA-binding by RIBEX (predicted RBPs).

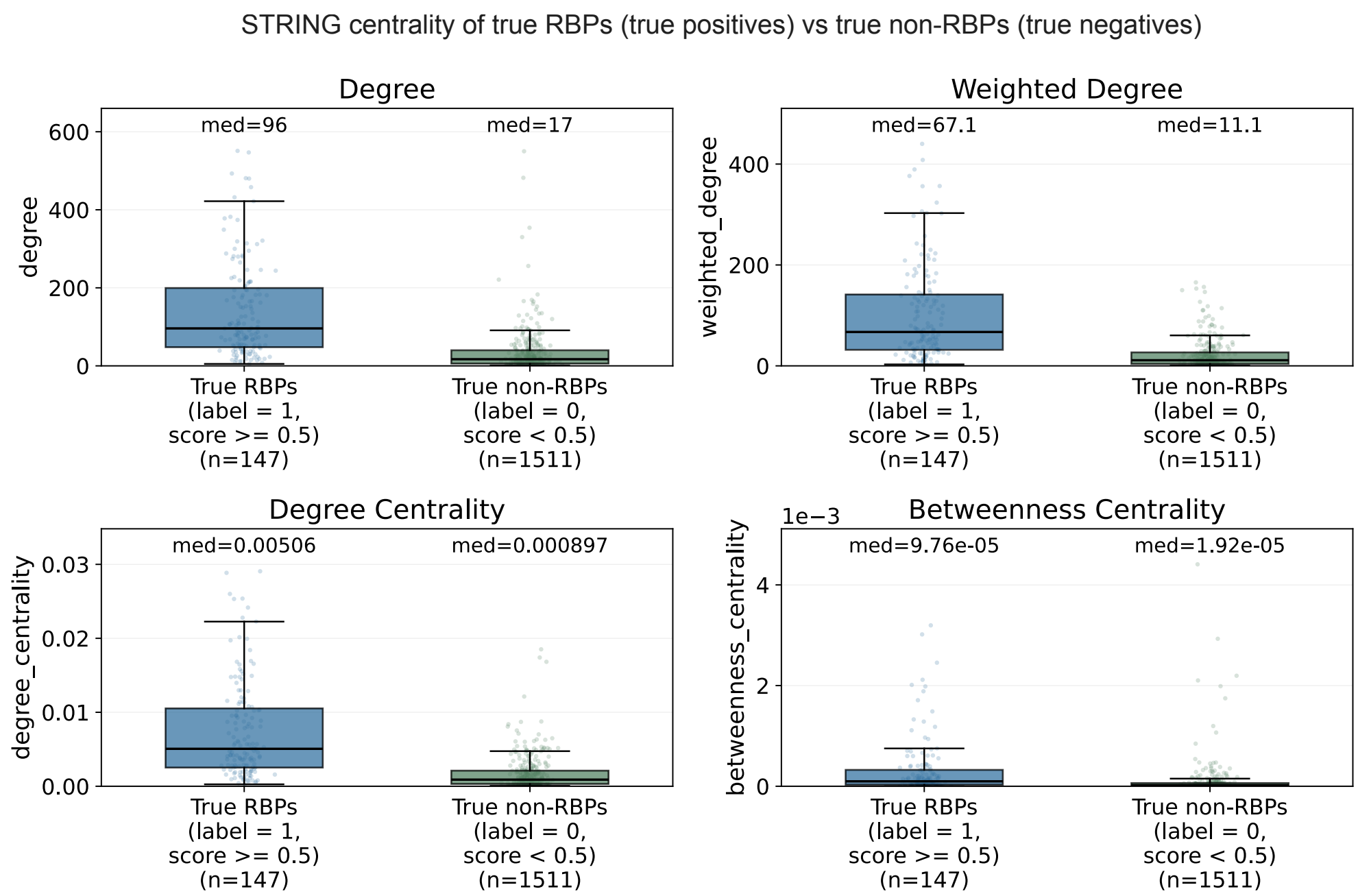

Figure 10 Centrality comparison of true RBPs vs. true non-RBPs. Network centrality measures are compared between true-positive predictions (correctly identified RBPs) and true-negative predictions (correctly identified non-RBPs).

C. Interpretability Analyses

C.1. Structural Support for Alanine-Scan Attributions

To provide an orthogonal check on the biological meaning of the alanine-scan scores, we compared residue-level perturbation scores with the distance from each corresponding residue to the closest RNA molecule in available protein–RNA structures from the PDB. More negative alanine-scan scores correspond to sequence windows whose perturbation lowers the predicted RNA-binding probability. These scores were enriched at shorter residue-to-RNA distances, indicating that residues with stronger positive contribution to the model prediction tend to lie closer to RNA and are therefore more likely to participate in RNA-contacting or RNA-proximal interfaces.

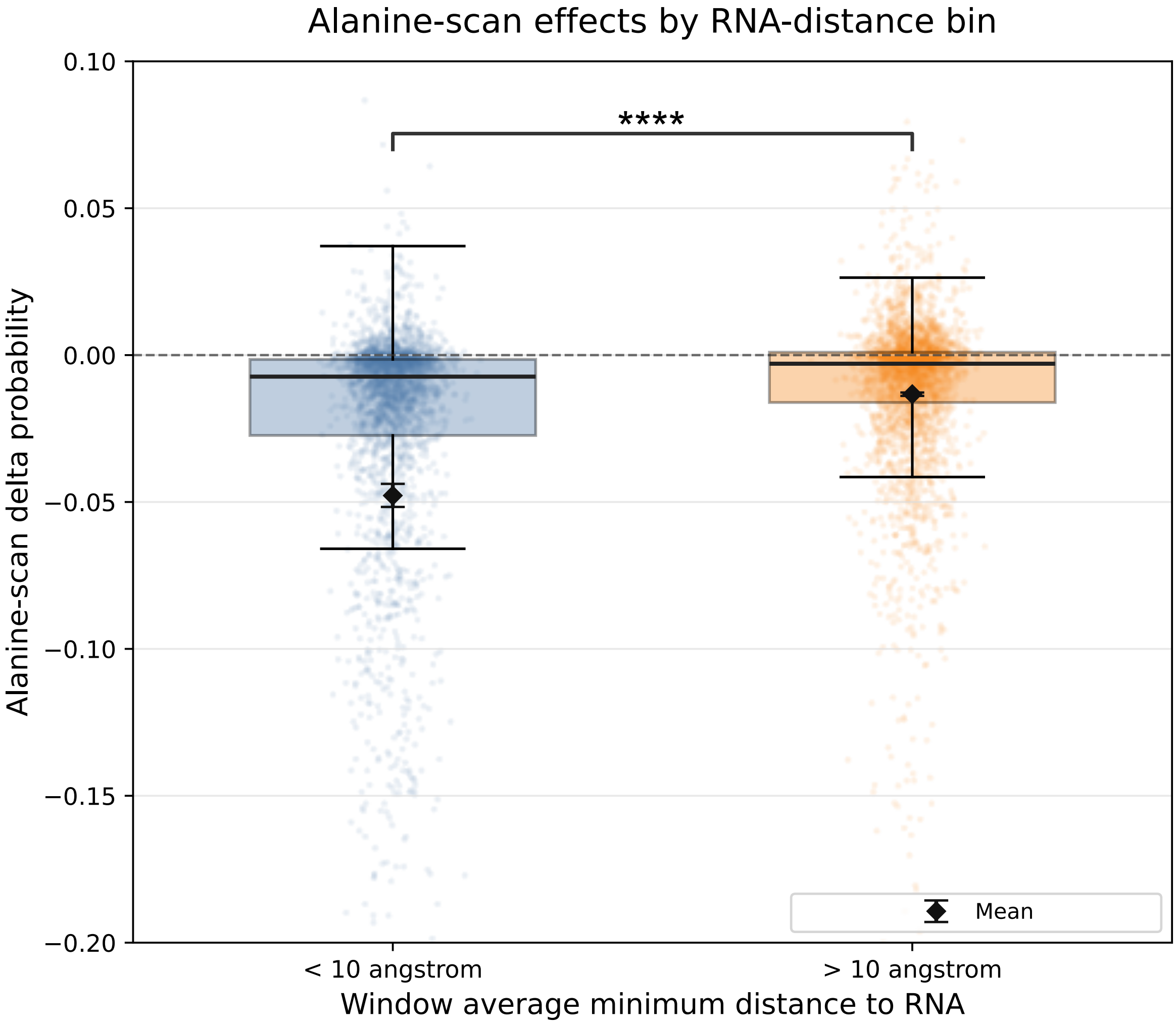

Figure 11 Relationship between alanine-scan attribution and RNA proximity in protein–RNA structures. Alanine-scan scores are compared with the distance from the corresponding residue to the closest RNA molecule reported in PDB structures. More negative scores are associated with lower residue-to-RNA distances, supporting the interpretation that strong negative perturbation scores identify residues or local sequence windows that are RNA-proximal and more likely to contribute to RNA-binding interfaces.

C.2. Literature-Anchored Alanine-Scan Case Studies

The revised main figure includes three case studies selected to connect the alanine-scan profiles to well-characterized RNA-binding biology. For LIN28A (Q9H9Z2), the strongest N-terminal perturbation occurs for windows beginning around positions ∼25–30, which span the boundary between an N-terminal disordered region and the cold-shock domain. Structural studies support the cold-shock and zinc-knuckle domains as the main pre-let-7 recognition and remodeling modules (Nam et al. 2011, Mayr et al. 2012); therefore, we interpret the N-terminal signal conservatively as a possible RNA-binding-context or remodeling contribution, not as direct evidence that this short N-terminal segment forms an experimentally validated RNA-contact site. This example illustrates a case in which disordered sequence context can cooperate with structured domains during RNA recognition, rather than acting as an isolated canonical RBD.

For PUM2, residue-level perturbation recapitulates the expected importance of the C-terminal PUM-HD domain, the canonical sequence-specific RNA-binding domain of human PUMILIO proteins (Lu and Tanaka Hall 2011). Additional importance is observed in the N-terminal disordered region, including residues overlapping the experimentally defined RD2 segment. Because the major direct RNA-binding activity of PUM2 is attributed to the PUM-HD, we interpret the N-terminal signal as a potential regulatory or RBP-associated sequence feature rather than as evidence for a second canonical RNA-binding domain.

As a positive-control example with a compact and well-characterized RNA-binding architecture, we applied the alanine-scanning interpretation to TARDBP/TDP-43. The alanine-scan profile shows pronounced negative changes in predicted RBP probability across the annotated RRM1 and RRM2 regions, indicating that perturbing these residues reduces the model's confidence in the RBP classification. This pattern is consistent with the established role of the tandem RRM region in sequence-specific recognition of UG-rich RNA by TDP-43 (Lukavsky et al. 2013). The strongest and most coherent attribution signal therefore overlaps the known RNA-binding modules rather than being diffusely distributed across the protein sequence. Additional signal is observed in the N-terminal and low-complexity/disordered regions, but the recovery of both canonical RRMs provides a clear example in which the model explanation agrees with experimentally characterized RNA-binding biology.

C.3. IDR-rich RBP Alanine-Scan Examples

To complement the main Figure 3 examples, we also examined IDR-rich RBPs where RNA association is expected to involve low-complexity, basic, or RG/RGG-rich regions in addition to, or instead of, compact canonical domains. The corresponding alanine-scan profiles are shown in Figure 12, with emphasis on FUS, SERBP1, TIA1, and DDX4 as literature-supported examples of disordered RNA-associated regions.

FUS (P35637) is a highly intrinsically disordered RBP with a low-complexity prion-like N-terminal region and multiple RGG-rich regions. RNA binding nucleates higher-order FUS ribonucleoprotein assembly, and both the low-complexity and RGG-rich regions contribute to this assembly behaviour (Schwartz et al. 2013). The alanine-scan profile therefore provides an example where sensitivity in low-complexity/RGG-rich regions is biologically plausible for an RNP-granule-associated RBP.

SERBP1 (Q8NC51) is largely intrinsically disordered and lacks a classical structured RNA-binding domain, but contains RG/RGG-repeat regions. Structural and biophysical characterization showed that SERBP1 is primarily disordered and recognizes G-rich RNA through its C-terminal RGG box and neighbouring residues (Baudin et al. 2021). Thus, alanine-sensitive disordered/basic regions in SERBP1 are consistent with RNA interactions mediated through non-canonical disordered sequence features.

TIA1 (P31483) combines structured RRMs with a Q/N-rich prion-related IDR that promotes stress-granule assembly (Gilks et al. 2004). RNA binding can enhance TIA1 self-association and phase separation in a multivalent, binding-site-dependent manner (Loughlin et al. 2021). The alanine-scan profile is therefore interpreted as reflecting both RNA-recognition modules and the disordered assembly-promoting region that contributes to RNA granule biology.

DDX4 (Q9NQI0) contains an N-terminal RG/RGG-rich IDR that drives phase separation of germ-granule-like condensates (Nott et al. 2015). Because RG/RGG motifs are established mediators of nucleic-acid interactions (Thandapani et al. 2013), sensitivity in this region is consistent with RNA-contacting and condensate-driving contributions to DDX4/Vasa RNP biology.

AFF4 (Q9UHB7) is retained as an additional supporting IDR-rich example. AFF4 is an intrinsically disordered scaffold protein of the super elongation complex and contains a conserved C-terminal homology domain that mediates AFF-family dimerization and binds RNA and DNA *in vitro* (Chen and Cramer 2019). The strongest perturbation signal localizes to this C-terminal homology domain, while additional signal in disordered regions is interpreted as RNA-associated or complex-context evidence rather than direct proof of RNA contact.

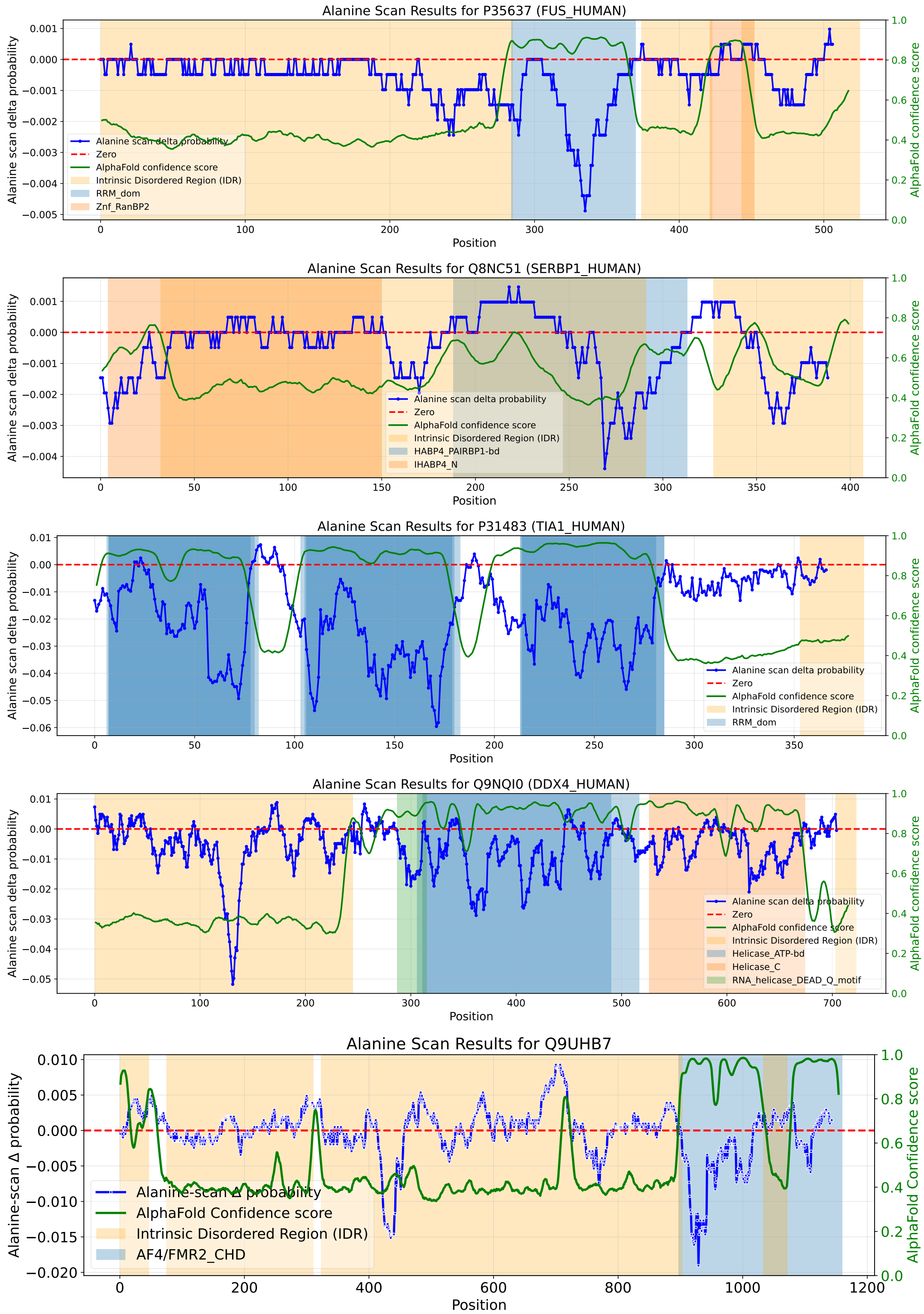

Figure 12 Alanine-scan profiles for IDR-rich RBPs. Profiles are shown for FUS (P35637), SERBP1 (Q8NC51), TIA1 (P31483), DDX4 (Q9NQI0), and AFF4 (Q9UHB7). These examples illustrate how RIBEX can assign predictive importance to low-complexity, basic, or RG/RGG-rich regions that participate in RNA binding, RNP assembly, or RNA-granule biology.

C.4. Additional Alanine Scan Results

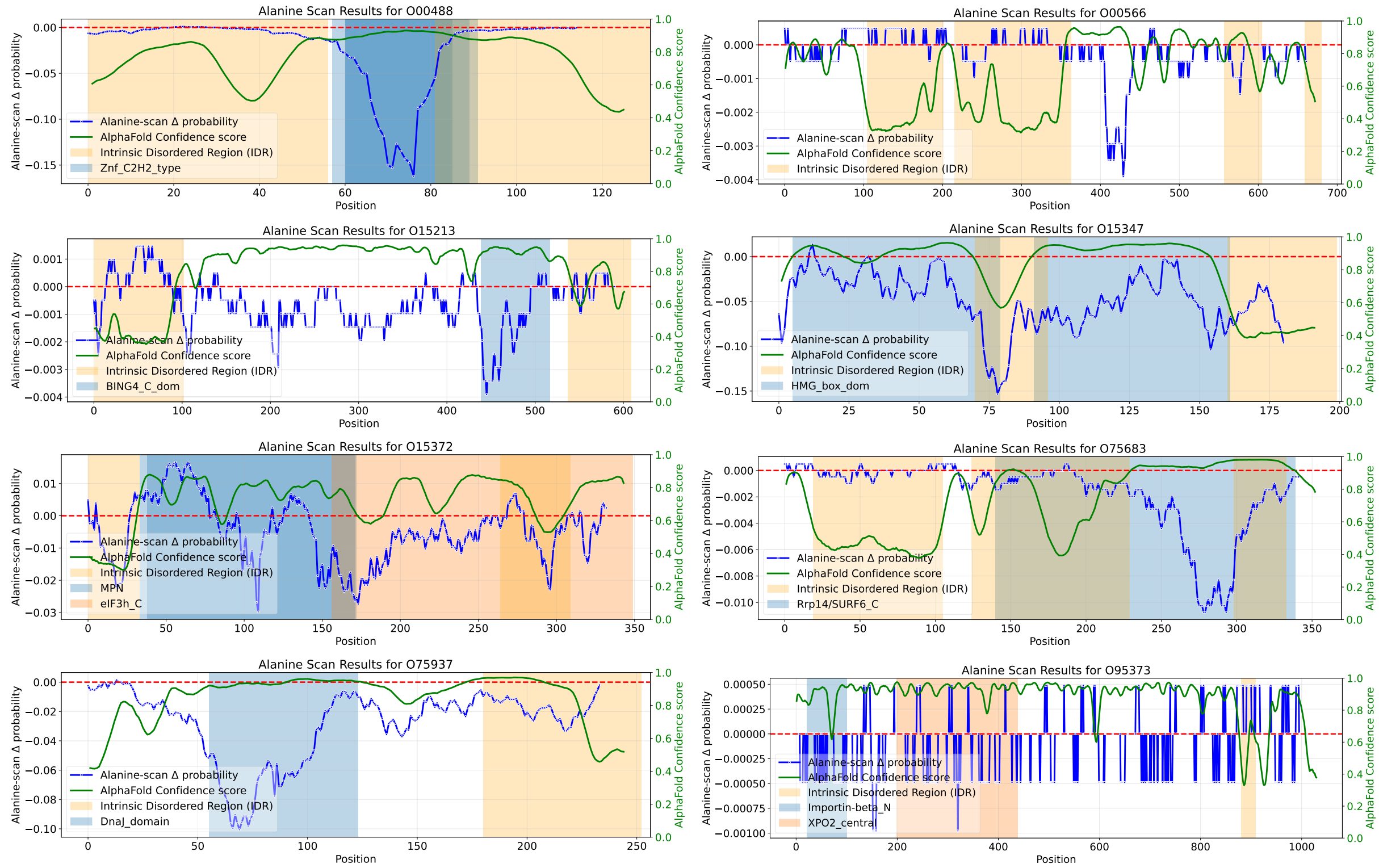

Figure 13 Alanine-scan attribution maps for selected proteins. Each panel shows the alanine-scan–based importance profile for the corresponding UniProt ID. The figure continues on the following pages.

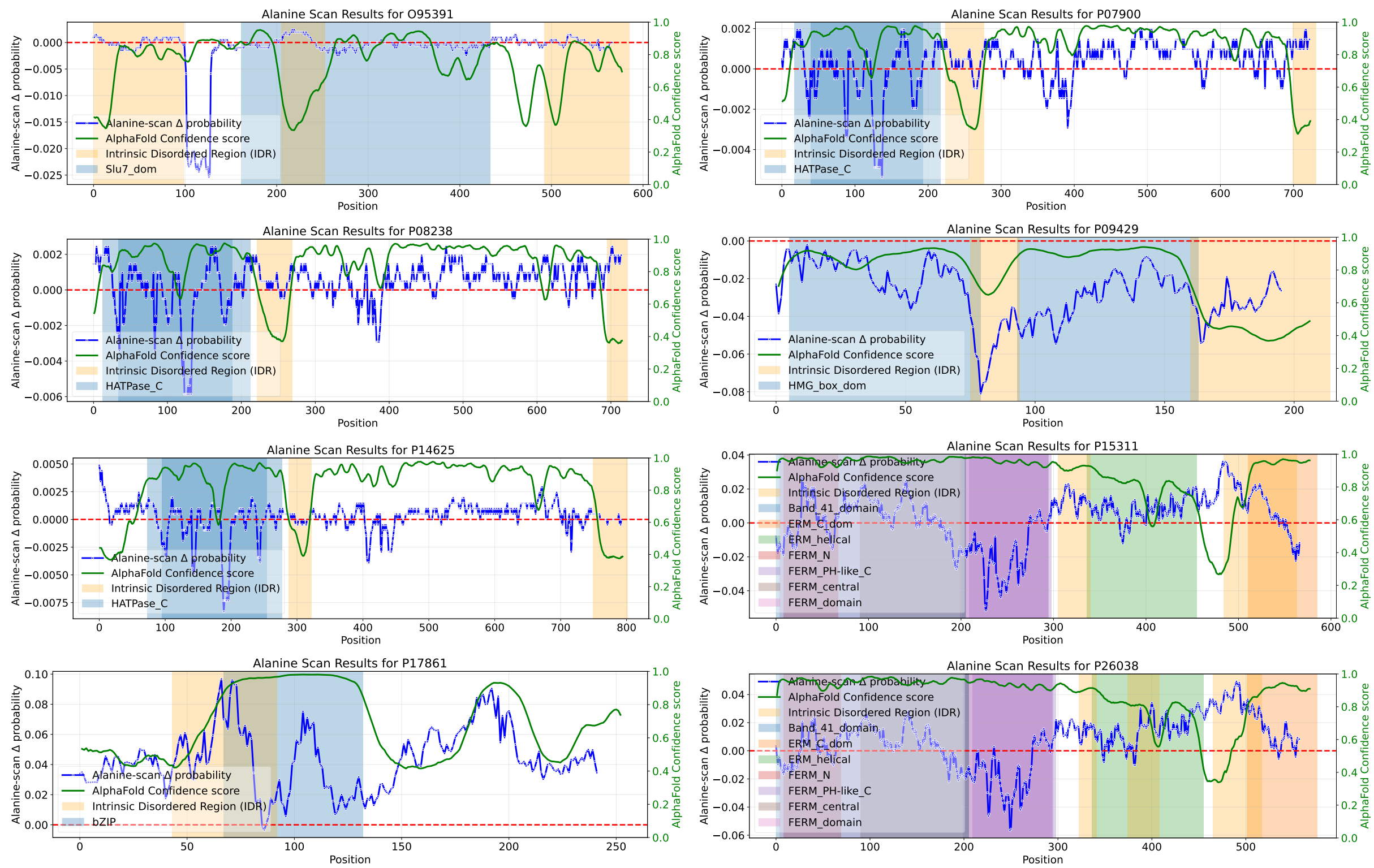

Figure 14 Alanine-scan attribution maps for selected proteins (continued).

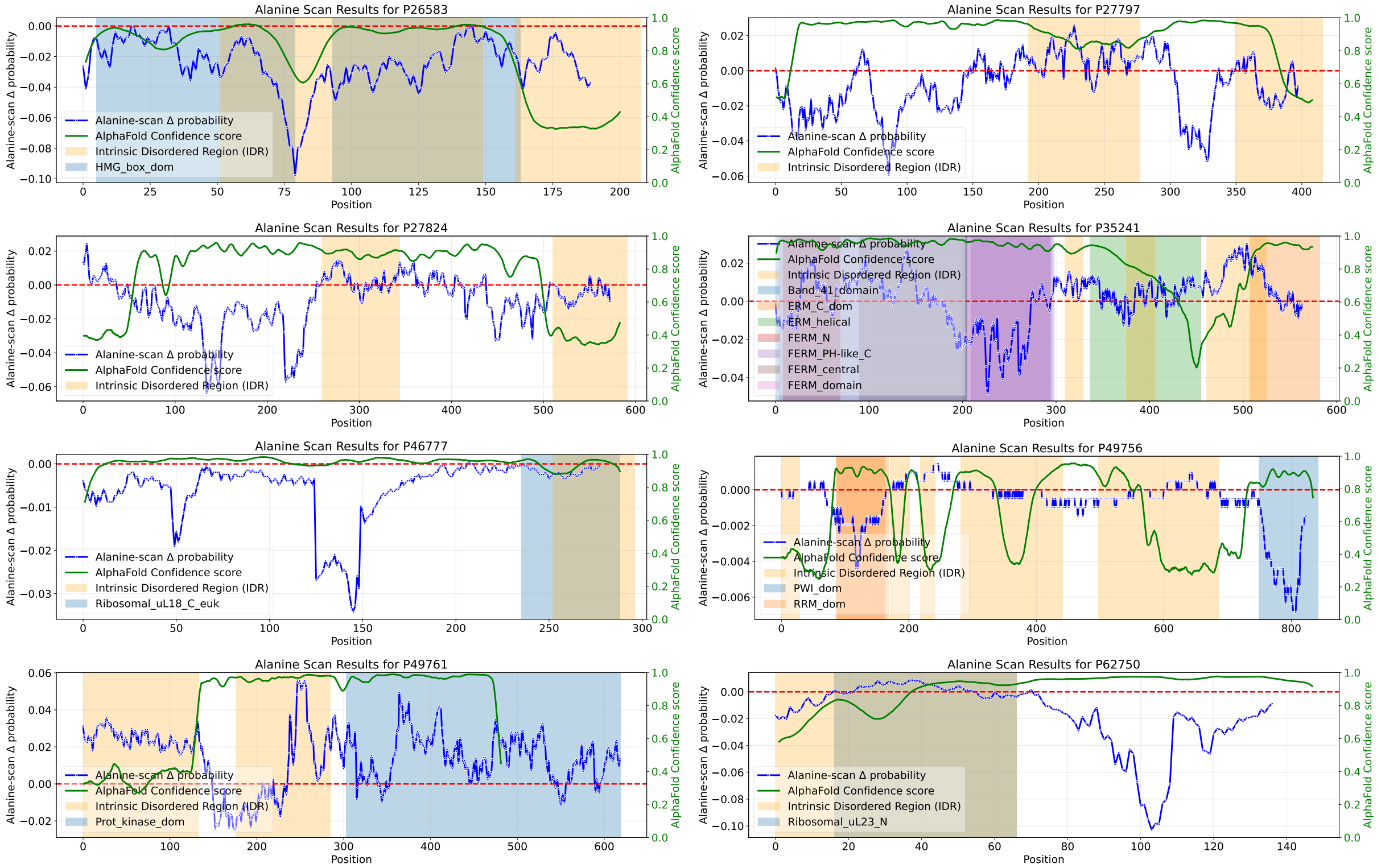

Figure 15 Alanine-scan attribution maps for selected proteins (continued).

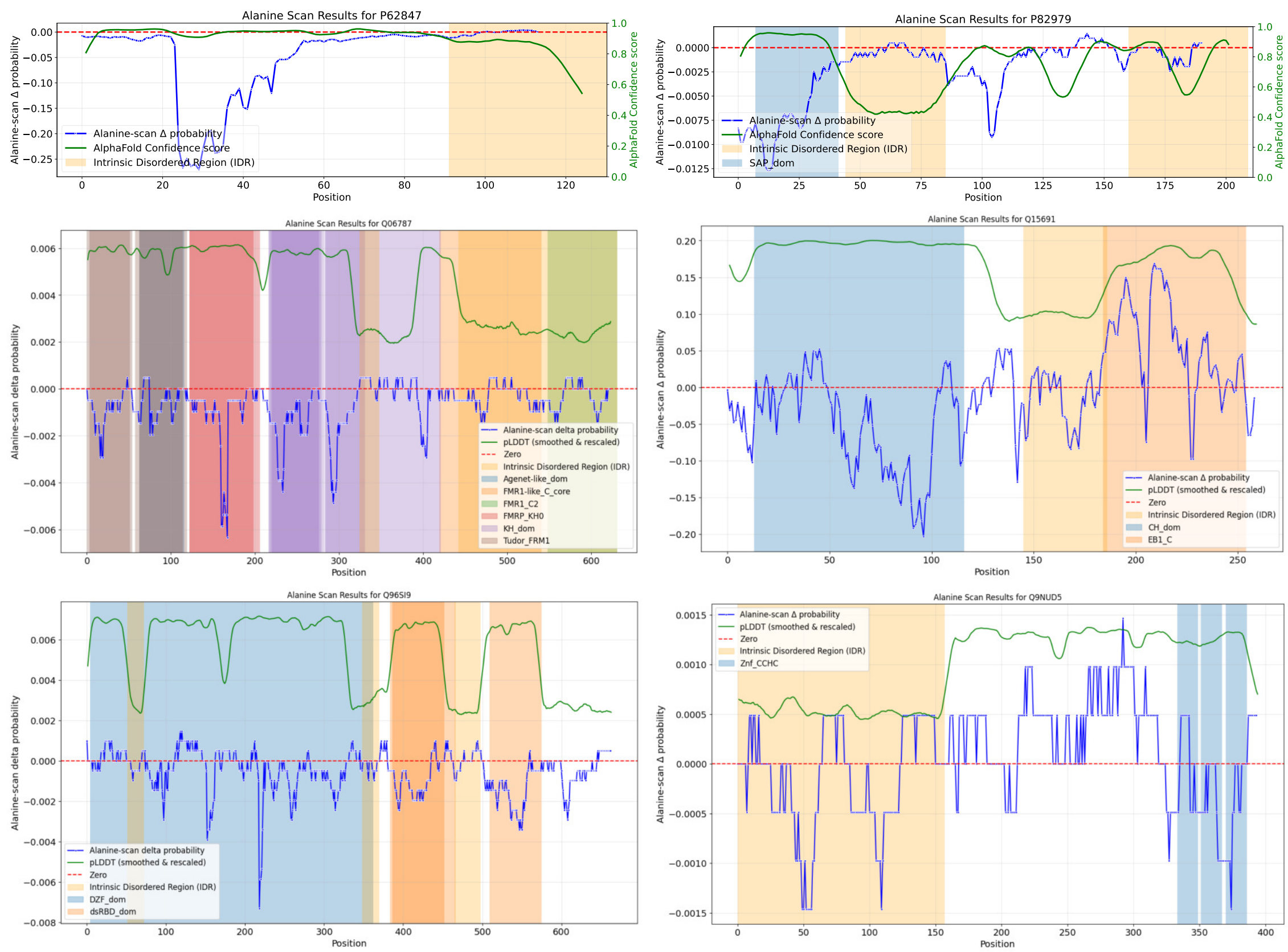

Figure 16 Alanine-scan attribution maps for selected proteins (continued).

Additional alanine-scan examples.

The previous main-text examples Q9UGR2 and HMGB1 are retained here as additional supporting cases. The alanine scan of **Q9UGR2**, a regulator of miRNA biogenesis, shows a sharply localized perturbation overlapping the annotated CCCH-type zinc-finger RNA-binding domain (ZnF_CCCH), while flanking regions display minimal effects, indicating that the model concentrates its sequence-level evidence on this compact RNA-binding module. In **P09429 (HMGB1)**, a multifunctional chromatin-associated protein with RNA-binding activity, the alanine scan reveals the largest drop in predictive probability at the IDR boundary between the A-box and the adjacent hinge (∼70–90), with additional sensitivity in the unstructured acidic tail (aa 186–215). Although HMGB1 is known to bind structured RNAs (Sofiadis et al. 2021), precise interaction sites remain unclear; this disordered segment may instead help position the two HMG boxes for coordinated RNA binding rather than contacting RNA directly.

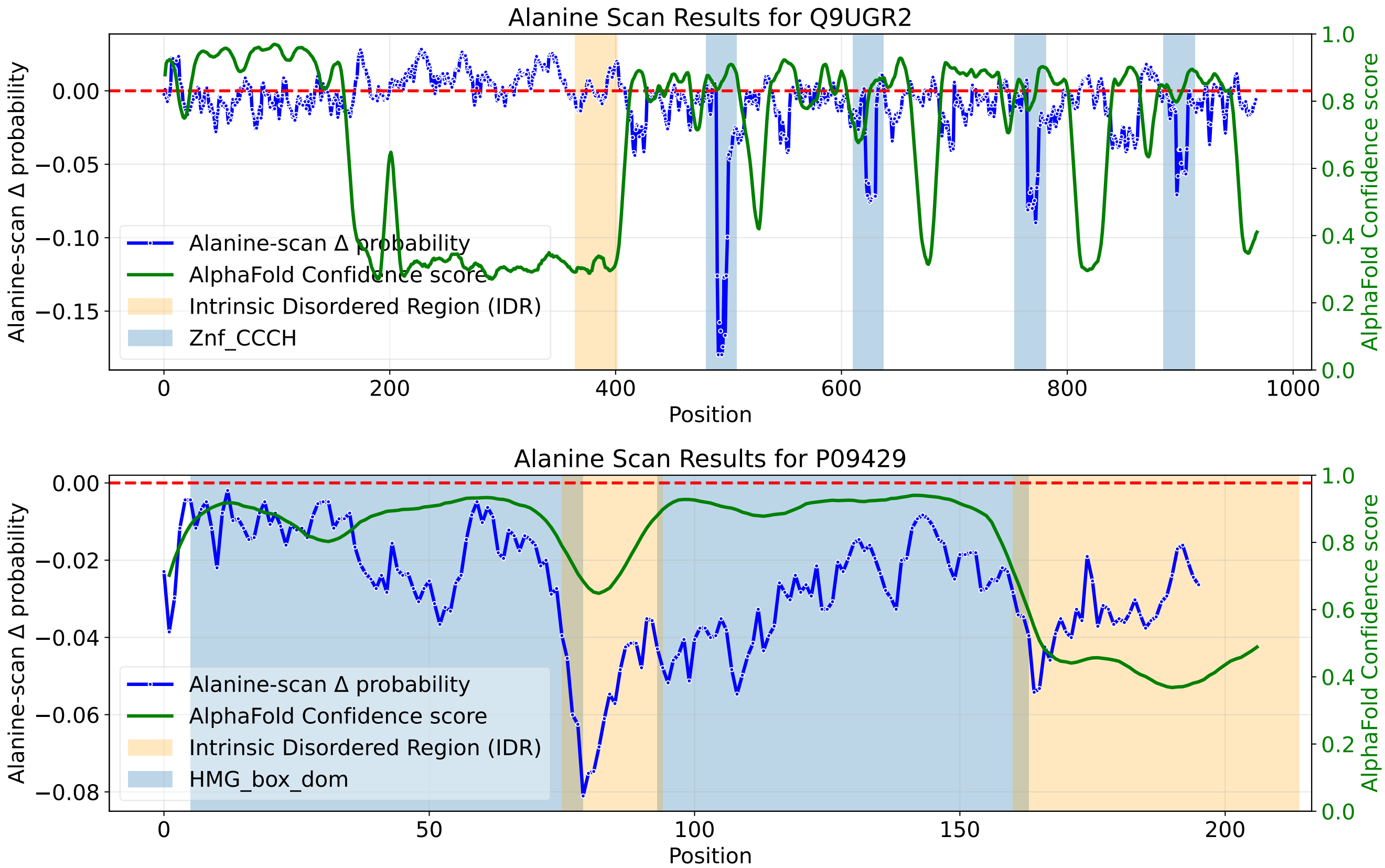

Figure 17 Additional alanine-scan attribution examples moved from the main text. Q9UGR2 shows a localized perturbation over a compact CCCH-type zinc-finger RNA-binding domain, whereas HMGB1 highlights a broader sensitivity pattern involving structured HMG boxes and adjacent disordered regions.

Additional HMGB-family examples.

Beyond the HMGB1 example retained in Figure 17, the attribution maps in Figures 13 and 18 show qualitatively similar profiles for P26583 (HMGB2) and O15347 (HMGB3). In both cases, the signal extends across the flexible linker/IDR context and into the acidic C-terminal tail, consistent with a broader contribution of disordered inter-domain regions to the model's RNA-associated predictions. These related profiles support the interpretation that, across HMGB-family proteins, such segments can shape the prediction without necessarily corresponding to direct RNA-contacting motifs.

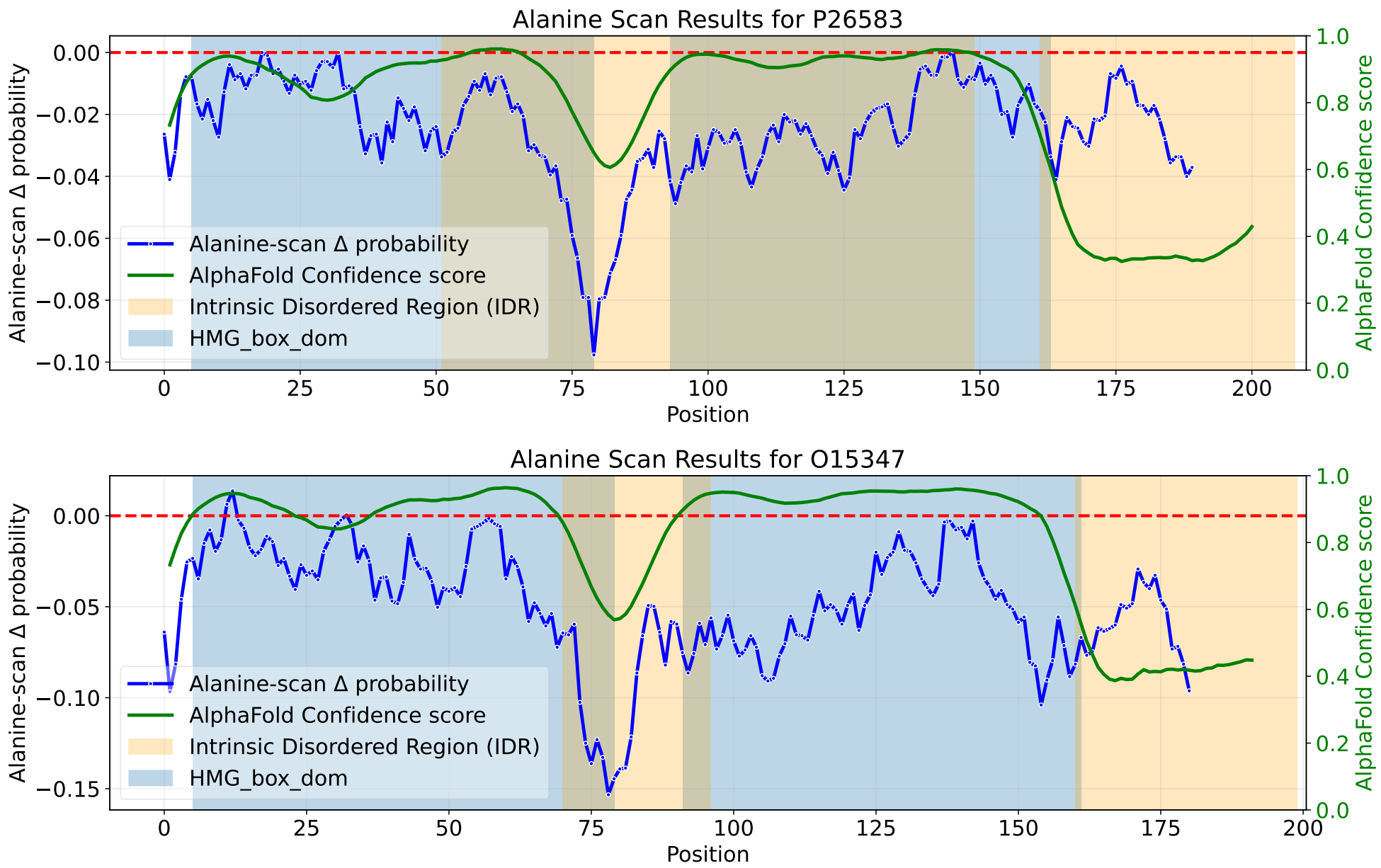

Figure 18 Focused HMGB-family alanine-scan attribution maps. The attribution profiles for P26583 (HMGB2; left) and O15347 (HMGB3; right) highlight signal spanning flexible linker/IDR context and the acidic C-terminal region, complementing the HMGB1 example retained in Figure 17.

C.5. Supplementary Structural Hypothesis for BUD13 (Q9BRD0)

As an additional supplementary example, human BUD13 (Q9BRD0) shows a localized alanine-scan sensitivity around residues ∼580–590. Here we show the corresponding attribution profile and provide a structural interpretation as a hypothesis, not a residue-level assignment.

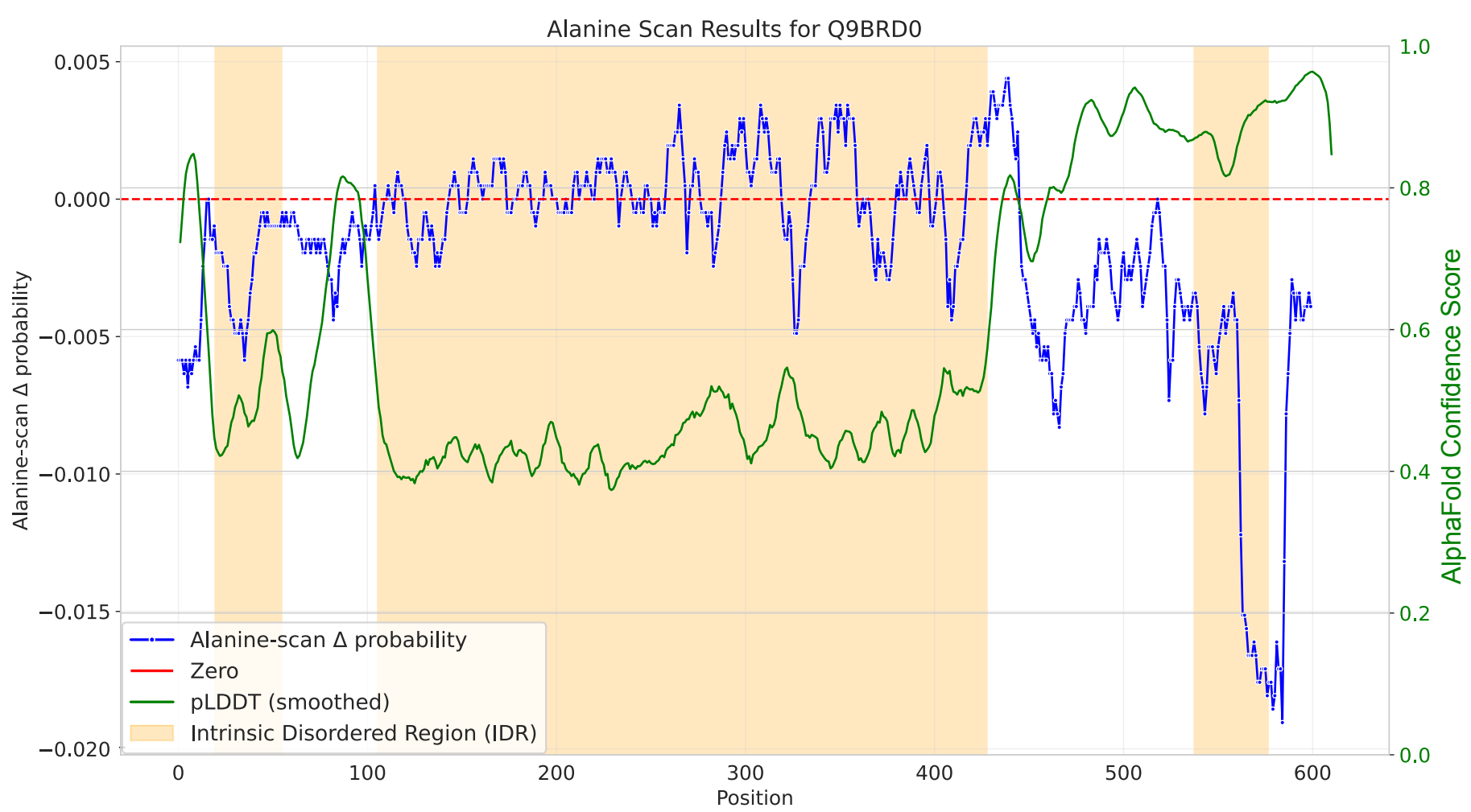

Figure 19 BUD13 (Q9BRD0) alanine-scan attribution profile. The strongest signal localizes around residues ∼580–590 in an annotated IDR, consistent with a candidate partner-binding segment in the RES-complex context.

By analogy to the yeast Snu17p–Bud13p RES-subcomplex structure (PDB 4UQT), in which a short Bud13p peptide binds the Snu17p RRM/UHM-like surface through a compact protein–protein interface (Tripsianes et al. 2014), the human BUD13 signal is compatible with a partner-binding segment involved in RES-complex assembly. This is also consistent with the signal falling in an annotated IDR with locally elevated pLDDT relative to the surrounding sequence. As an additional motif-level observation, an `RWDGV` sequence is present in human BUD13 near residues 583–587, matching a motif reported for the yeast ligand. This correspondence is suggestive, but it does not establish residue-level equivalence or conserved binding geometry.

References

RBPbase: a comprehensive database of eukaryotic RNA-binding proteins. `https://apps.embl.de/rbpbase/`, 2026. Version v0.23 alpha (accessed 13 February 2026).

M. Ashburner et al. Gene Ontology: tool for the unification of biology. *Nature Genetics*, 25(1):25–29, May 2000.

A. Bateman et al. The Pfam protein families database. *Nucleic Acids Research*, 32(Database issue):D138–141, Jan. 2004.

A. Baudin et al. Structural characterization of the RNA-binding protein SERBP1 reveals intrinsic disorder and atypical RNA binding modes. *Frontiers in Molecular Biosciences*, 8:744707, 2021.

D. Binns et al. QuickGO: a web-based tool for Gene Ontology searching. *Bioinformatics*, 25(22):3045–3046, Nov. 2009.

K. W. Brannan et al. SONAR discovers RNA-binding proteins from analysis of large-scale protein–protein interactomes. *Molecular Cell*, 64(2):282–293, 2016.

A. Bressin et al. Tripepsvm: de novo prediction of rna-binding proteins based on short amino acid motifs. *Nucleic Acids Research*, 47(9): 4406–4417, 2019.

A. Calderone, L. Castagnoli, and G. Cesareni. mentha: a resource for browsing integrated protein-interaction networks. *Nature Methods*, 10(8):690–691, Aug. 2013.

Y. Chen and P. Cramer. Structure of the super-elongation complex subunit AFF4 C-terminal homology domain reveals requirements for AFF homo- and heterodimerization. *Journal of Biological Chemistry*, 294(27):10663–10673, 2019.

T. U. Consortium. Uniprot: the universal protein knowledgebase in 2025. *Nucleic Acids Research*, 53(D1):D609–D617, 01 2025.

S. Gerstberger, M. Hafner, and T. Tuschl. A census of human rna-binding proteins. *Nature Reviews Genetics*, 15(12):829–845, 2014.

N. Gilks et al. Stress granule assembly is mediated by prion-like aggregation of TIA-1. *Molecular Biology of the Cell*, 15(12):5383–5398, 2004.

S. Granneman et al. The human Imp3 and Imp4 proteins form a ternary complex with hMpp10, which only interacts with the U3 snoRNA in 60-80s ribonucleoprotein complexes. *Nucleic Acids Research*, 31(7):1877–1887, 2003.

M. W. Hentze et al. A brave new world of RNA-binding proteins. *Nature Reviews Molecular Cell Biology*, 19(5):327–341, May 2018.

S. Holm. A simple sequentially rejective multiple test procedure. *Scandinavian journal of statistics*, pages 65–70, 1979.

E. J. Hu et al. Lora: Low-rank adaptation of large language models. *arXiv preprint arXiv:2106.09685*, 2021.

E. L. Huttlin et al. The BioPlex Network: A Systematic Exploration of the Human Interactome. *Cell*, 162(2):425–440, July 2015.

W. Jin et al. HydRA: Deep-learning models for predicting RNA-binding capacity from protein interaction association context and protein sequence. *Molecular Cell*, 83(14):2595–2611.e11, 2023.

W. Li and A. Godzik. CD-HIT: a fast program for clustering and comparing large sets of protein or nucleotide sequences. *Bioinformatics*, 22(13):1658–1659, 2006.

L. Licata et al. MINT, the molecular interaction database: 2012 update. *Nucleic Acids Research*, 40(D1):D857–D861, Jan. 2012.

Z. Lin et al. Language models of protein sequences at the scale of evolution enable accurate structure prediction. *bioRxiv*, 2022.

F. E. Loughlin et al. Tandem RNA binding sites induce self-association of the stress granule marker protein TIA-1. *Nucleic Acids Research*, 49(5):2403–2417, 2021.

G. Lu and T. M. Tanaka Hall. Alternate modes of cognate RNA recognition by human PUMILIO proteins. *Structure*, 19(3):361–367, 2011.

P. J. Lukavsky et al. Molecular basis of UG-rich RNA recognition by the human splicing factor TDP-43. *Nature Structural & Molecular Biology*, 20:1443–1449, 2013.

F. Mayr et al. The Lin28 cold-shock domain remodels pre-let-7 microrna. *Nucleic Acids Research*, 40(15):7492–7506, 2012.

Y. Nam et al. Molecular basis for interaction of let-7 micrornas with Lin28. *Cell*, 147(5):1080–1091, 2011.

T. J. Nott et al. Phase transition of a disordered nuage protein generates environmentally responsive membraneless organelles. *Molecular Cell*, 57(5):936–947, 2015.

L. Page et al. The pagerank citation ranking: Bringing order to the web. Technical report, Stanford InfoLab, 1999.

X. Peng et al. Rbp-tstl is a two-stage transfer learning framework for genome-scale prediction of rna-binding proteins. *Briefings in Bioinformatics*, 23(4):bbac215, 2022.

E. Perez et al. Film: Visual reasoning with a general conditioning layer. In *Proceedings of the AAAI Conference on Artificial Intelligence*, volume 32, pages 3942–3951, 2018.

A. Schultz et al. Protein-protein and protein-rna contacts both contribute to the 15.5k-mediated assembly of the u4/u6 snrnp and the box c/d snornps. *Molecular and Cellular Biology*, 26(13):5146–5154, 2006.

J. C. Schwartz et al. RNA seeds higher-order assembly of FUS protein. *Cell Reports*, 5(4):918–925, 2013.

K. Sofiadis et al. Hmgb1 coordinates sasp-related chromatin folding and rna homeostasis on the path to senescence. *Molecular Systems Biology*, 17(6):e9760, 2021.

M. Steinegger and J. Söding. Mmseqs2 enables sensitive protein sequence searching for the analysis of massive data sets. *Nature Biotechnology*, 35(11):1026–1028, 2017.

D. Szklarczyk et al. The string database in 2023: protein–protein association networks and functional enrichment analyses for any sequenced genome of interest. *Nucleic Acids Research*, 51(D1):D638–D646, 2023.

P. Thandapani et al. Defining the RGG/RG motif. *Molecular Cell*, 50(5):613–623, 2013.

The Gene Ontology Consortium. The Gene Ontology knowledgebase in 2026. *Nucleic Acids Research*, 54(D1):D1779–D1792, Jan. 2026.

K. Tripsianes et al. A novel protein–protein interaction in the RES (REtention and Splicing) complex. *Journal of Biological Chemistry*, 289(41):28640–28650, 2014.

C. Wang and U. T. Meier. Architecture and assembly of mammalian H/ACA small nucleolar and telomerase ribonucleoproteins. *The EMBO Journal*, 23(8):1857–1867, 2004.

N. J. Watkins et al. A common core RNP structure shared between the small nucleolar box C/D RNPs and the spliceosomal U4 snRNP. *Cell*, 103(3):457–466, 2000.
